# Living Therapeutics for Synergistic Hydrogen‐Photothermal Cancer Treatment by Photosynthetic Bacteria

**DOI:** 10.1002/advs.202408807

**Published:** 2024-11-04

**Authors:** Yingyi Zhang, Xiaolian Deng, Lili Xia, Jianghui Liang, Meng Chen, Xiaoling Xu, Wei Chen, Jianwei Ding, Chengjie Yu, Limei Liu, Yang Xiang, Yiliang Lin, Fangfang Duan, Wei Feng, Yu Chen, Xiang Gao

**Affiliations:** ^1^ Department of Pharmacology School of Medicine Shenzhen Campus of Sun Yat‐Sen University Shenzhen 518107 P. R. China; ^2^ Key Laboratory of Quantitative Synthetic Biology Shenzhen Institute of Synthetic Biology Shenzhen Institutes of Advanced Technology Chinese Academic of Science Shenzhen 518000 P. R. China; ^3^ Materdicine Lab School of Life Sciences Shanghai University Shanghai 200444 P. R. China; ^4^ Department of General Surgery Sir Run Run Shaw Hospital School of Medicine Zhejiang University Zhejiang 310016 P. R. China; ^5^ Key laboratory for accurate diagnosis and treatment of abdominal infection in Zhejiang province Sir Run Run Shaw Hospital School of Medicine Zhejiang University Zhejiang 310016 P. R. China; ^6^ Department of Chemical and Biomolecular Engineering National University of Singapore Singapore 117585 Singapore

**Keywords:** hydrogen therapy, hydrogen‐photothermal therapy, living bacterial therapies, photosynthetic bacteria

## Abstract

Hydrogen gas (H_2_) therapy, recognized for its inherent biosafety, holds significant promise as an anti‐cancer strategy. However, the efficacy of H_2_ treatment modalities is compromised by their reliance on systemic gas administration or chemical reactions generation, which suffers from low efficiency, poor targeting, and suboptimal utilization. In this study, living therapeutics are employed using photosynthetic bacteria *Rhodobacter sphaeroides* for in situ H_2_ production combined with near‐infrared (NIR) mediated photothermal therapy. Living *R. sphaeroides* exhibits strong absorption in the NIR spectrum, effectively converting light energy into thermal energy while concurrently generating H_2_. This dual functionality facilitates the targeted induction of tumor cell death and substantially reduces collateral damage to adjacent normal tissues. The findings reveal that integrating hydrogen therapy with photothermal effects, mediated through photosynthetic bacteria, provides a robust, dual‐modality approach that enhances the overall efficacy of tumor treatments. This living therapeutic strategy not only leverages the therapeutic potential of both hydrogen and photothermal therapeutic modalities but also protects healthy tissues, marking a significant advancement in cancer therapy techniques.

## Graphical abstract

The development of hydrogen gas (H_2_) therapy is highly challenging by reason of low efficiency and suboptimal utilization due to its high diffusibility, nonpolarity, and low solubility of H_2_ in physiological conditions, which prevent long‐term release and reduce therapeutic efficiency. In light of these challenges, the research offers a novel approach that uses living photosynthetic bacteria, *R. sphaeroides*, to generate and release H_2_ for enhanced effective time under the control of NIR light, and also converts NIR light to thermal energy efficiently. This strategy providing a synergistic effect that significantly enhances tumor suppression while protecting normal tissues, which makes it a distinct candidate for anti‐tumor therapies.

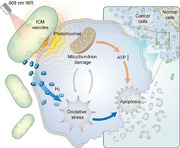

## 1 Introduction

Cancer continues to pose the most significant health threat globally, but traditional treatments such as chemotherapy and radiotherapy often demonstrate limited efficacy and substantial non‐specific toxicity to normal tissues, thereby inflicting considerable discomfort on patients.^[^ ^1^ ^]^ Rapid advancements in nanobiotechnology have led to the development of a variety of therapeutic platforms that enhance the precision and effectiveness of cancer treatment.^[^ ^2^ ^]^ These platforms facilitate the development of versatile nanomedicines, including gas‐based therapeutics that specifically eliminate cancer cells while protecting healthy cells, thereby minimizing the adverse effects commonly associated with conventional cancer therapies.^[^ ^3^ ^]^ Several types of physiologically gaseous molecules such as oxygen (O_2_),^[^ ^4^ ^]^ nitric oxide (NO),^[^ ^5^ ^]^ hydrogen sulfide (H_2_S),^[^ ^6^ ^]^ carbon monoxide (CO),^[^ ^7^ ^]^ sulfur dioxide (SO_2_), and hydrogen gas (H_2_)^[^ ^8^ ^]^ have the function of regulating vasodilatation, neurotransmission, anti‐inflammatory, and anti‐oxidative reactions in both physiological and pathophysiological processes. Its specific therapeutic effects have been documented in diseases ranging from cardiovascular ailments to neurodegenerative disorders and cancers, thanks to its ability to modulate physiological functions.

H_2_, the lightest molecule in nature, constitutes ≈0.5 parts per million of the Earth's atmosphere. Research dating back to 1975 has highlighted the potential of H_2_ in cancer treatment, particularly due to its selectively anti‐oxidative properties, which allows for the modulation of reactive oxygen species (ROS) levels within cancer cells.^[^ ^9^ ^]^ Currently, hydrogen therapy excels over other gas‐based treatments due to its selective antioxidative properties, excellent safety profile, broad therapeutic applications, synergistic potential with other therapies, and minimal disruption to normal cellular processes.^[^ ^10^ ^]^ However, current H_2_ delivery methods are limited by its high diffusibility, nonpolarity, and low solubility in physiological conditions, which prevent long‐term release and reduce therapeutic efficiency.^[^ ^11^ ^]^ In situ H_2_ generation technologies, such as self‐decomposition of nanosystems and light‐mediated water splitting, often suffer from low catalytic efficiency.^[^ ^12^ ^]^ Addressing these delivery and long‐term in situ generation challenges is crucial for maximizing the clinical benefits of hydrogen therapy. Moreover, the integration of hydrogen therapy with conventional treatment modalities like chemotherapy and radiotherapy has demonstrated significant synergistic therapeutic effects.^[^ ^13^ ^]^ Biohydrogen production offers sustainable, environmentally friendly, and efficient energy generation from renewable energy, providing versatile and economically viable application,^[^ ^14^ ^]^ including the potential for long‐term hydrogen release to enhance therapeutic efficiency.^[^ ^15^ ^]^ Photosynthetic bacteria, such as purple non‐sulfur bacteria (PNSB), are favored for their ability to efficiently produce and release H_2_ under illumination via hydrogenase and their capacity to metabolize a wide range of substrates,^[^ ^16^ ^]^ which is potentially act as living therapeutics. The advantages of living therapeutics, particularly in terms of efficacy, precise control, and safety, highlight its transformative potential to redefine and expand the scope of future therapeutic strategies. This innovative approach offers the ability to harness living systems for more targeted, adaptive, and sustainable treatments, positioning it as a pivotal advancement in the next generation of medicine.^[^ ^17^ ^]^

**Figure 1:**
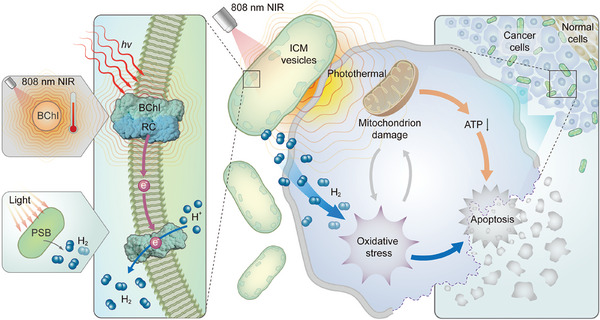
Schematic illustration showing the process of biohydrogen production and photothermal conversion of *R. sphaeroides* for synergistic hydrogen‐photothermal therapy. BChl, bacteriochlorophyll; RC, Reaction center; PSB, Photosynthetic bacteria; ICM, Intracytoplasmic membrane.

In this work, we develop living therapeutics using photosynthetic bacteria, *Rhodobacter sphaeroides*, for long‐term H_2_ production coupled with hyperthermia induction under near‐infrared (NIR) light irradiation for tumor hydrogen‐photothermal therapy. *R. sphaeroides*, a facultative anaerobic bacterium, is well‐suited for growth in hypoxic conditions and demonstrates phototaxis.^[^ ^18^ ^]^ These unique traits enhance its ability to adapt to the tumor microenvironment and migrate toward illuminated areas, making it an ideal candidate for tumor targeting and therapy. Living *R. sphaeroides* excels as photosynthetic hydrogen producers and show remarkable NIR absorption, efficient conversion of NIR light to heat, high photothermal stability, with negligible cytotoxic effects. Consequently, we hypothesize that the *R. sphaeroides* can simultaneously produce hydrogen gas and generate heat under 808 nm laser irradiation, enabling a dual‐mode therapy that combines hydrogen production with photothermal effects. Utilizing this bacterium as a dual supplier for hydrogen and photothermal effects allows for more precise and controllable delivery of hydrogen, while also demonstrating relatively high biocompatibility. Importantly, *R. sphaeroides*‐mediated synergistic hydrogen‐photothermal therapy has no discernible impact on normal cells, underscoring its potential to protect healthy normal cells from hyperthermia damage while selectively targeting tumor cells. Further investigations reveal that this synergistic strategy notably increases ROS generation within cancer cells, disrupting their redox homeostasis and impairing mitochondrial function, which ultimately leads to apoptosis (**Figure** **1**). By utilizing the inherent biological capabilities of *R. sphaeroides*, this study offers a potentially efficient living therapeutic strategy to mitigate tumor progression while safeguarding healthy cells. This dual‐action strategy not only targets malignant cells with precision but also minimizes collateral damage to surrounding non‐cancerous tissues, thus presenting a promising avenue for advancing cancer therapy.

## 2 Results and Discussions

### 2.1 Combined Hydrogen Production and Photothermal Effects of R. Sphaeroides

**Figure 2:**
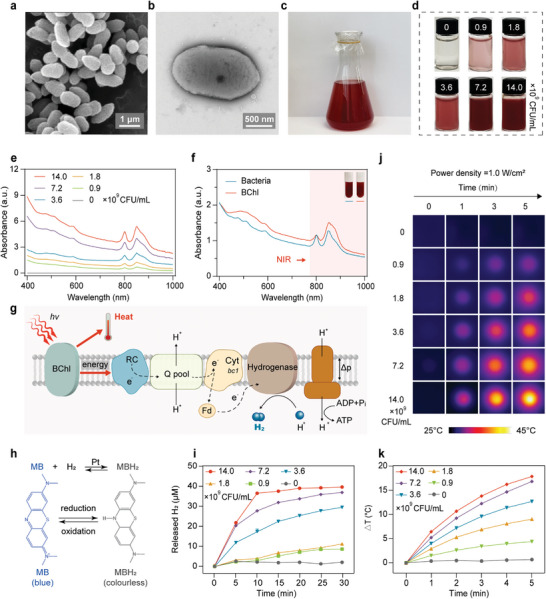
Characterization of *R. sphaeroides*. a) SEM image of the *R. sphaeroides*. b) TEM image of the *R. sphaeroides*. c) Photograph of the *R. sphaeroides* cultured in LB medium. d) Photographs of the *R. sphaeroides* suspended in PBS at different concentrations. e) Absorption spectra of the *R. sphaeroides* at different concentrations (0, 0.9×10^9^, 1.8×10^9^, 3.6×10^9^, 7.2×10^9^, and 1.4×10^10^ CFU mL^−1^). f) Absorption spectra of the *R. sphaeroides* and its BChl. g) Schematic illustrating the process of H_2_ production and photothermal conversion in *R. sphaeroides* under NIR light irradiation. (Q pool, Ubiquinone pool; Fd, Ferredoxin; Cyt bc1, Cytochrome bc1; Δp, Proton motive force; Pi, Inorganic phosphate). h) Schematic representation of H_2_ production as monitored using MB as the probe. i) H_2_ released from *R. sphaeroides* suspensions at different concentrations (0, 0.9×10^9^, 1.8×10^9^, 3.6×10^9^, 7.2×10^9^, and 14×10^9^ CFU mL^−1^) under irradiation of an 808 nm LED at 0.06 W cm^−2^ for 30 min (*n* = 3 biologically independent samples). j) Infrared thermal images of *R. sphaeroides* suspensions at different concentrations exposed to an 808 nm laser at 1.0 W cm^−2^ for 5 min, with an initial temperature at 27 °C. k) Photothermal temperature curves of *R. sphaeroides* suspensions at different concentrations (0, 0.9×10^9^, 1.8×10^9^, 3.6×10^9^, 7.2×10^9^, and 14×10^9^ CFU mL^−1^) under 808 nm laser irradiation at the power density of 1.0 W cm^−2^ for 5 min (*n* = 3 biologically independent samples). Data are presented as means ± standard deviation (SD).

*R. sphaeroides*, the most widely used model of purple nonsulfur photosynthetic bacteria, demonstrates versatile metabolic capabilities, including aerobic and anaerobic respiration, photosynthesis, and fermentation.^[^ ^18^ ^c]^ We cultured the *R. sphaeroides* in Luria Broth (LB) medium and performed the characterization. Scanning electron microscopy (SEM) and transmission electron microscopy (TEM) revealed its ovoid shape with a diameter approximately in 2 µm (**Figure** **2**a,b). After cultured for 12 h in the LB medium, *R. sphaeroides* turned red color (Figure 2c). The UV‐vis spectra indicated a wide range of absorption with peaks at 808 nm and 850 nm (Figure 2e), characteristic absorption peak of bacteriochlorophyll (BChl). BChl functions as a light‐harvesting antenna to use light energy for photosynthesis.^[^ ^16^ ^]^ As shown in Figure 2d,e, the intensity of coloration and absorbance of the bacterial suspension were concentration‐dependent. To confirm the absorption property was derived from BChl, it was extracted from *R. sphaeroides* and its absorption spectra were analyzed, revealing absorption peaks in the NIR region align with those of *R. sphaeroides* (Figure 2f). Within the intracytoplasmic membrane (ICM), BChl absorbs photons and channels energy to the photosynthetic reaction center (Figure 2g), initialing the photosynthetic electron transfer chain, which generates high‐energy electrons subsequently utilized by hydrogenase.^[^ ^19^ ^]^ Hydrogenase captures electrons derived from ferredoxin, facilitating the reduction of protons translocated across the membrane by adenosine‐triphosphate (ATP) synthase, culminating in H_2_ production.^[^ ^20^ ^]^

To measure the capability of *R. sphaeroides* to produce H_2_ under the control of NIR light, we collected the *R. sphaeroides* cells and resuspended them into phosphate‐buffered saline (PBS) with various bacterial concentrations and illuminated them under 808 nm light emitting diode (LED) light (0.06 W cm^−2^). The methylene blue (MB) reduction assay was used to quantify the H_2_ production (Figure 2h). The released H_2_ production was enhanced with an increase in bacterial cell concentration (Figures S1 and S2, Supporting Information). The accumulated H_2_ production reached 38.9 µM after 30 min with the bacteria concentration of 1.4×10^10^ CFU mL^−1^ (Figure 2i), showing 4‐fold increase compared with bacteria concentration of 9.0×10^8^ CFU mL^−1^, and 17‐fold increase compared with the PBS control (Figure 2i). The maximum H_2_ released rate was achieved during the first 10 min with the cell concentration of 1.4×10^10^ CFU mL^−1^, and reached 1.3 µM min^−1^ and 0.3 µM min^−1^ with the cell concentrations of 1.4×10^10^ CFU mL^−1^ and 9.0×10^8^ CFU mL^−1^, respectively (Figure S3, Supporting Information). Those results indicated the *R. sphaeroides* could generate and release H_2_ efficiently under the control of NIR irradiation.

BChl exhibits an obvious absorption band in the 800 – 810 nm range, indicating *R. sphaeroides* could convert NIR light into thermal energy efficiently (Figure 2g). The photothermal conversion performance of *R. sphaeroides* was assessed by exposing different concentrations of bacterial cells to an 808 nm laser (1.0 W/cm^2^) and monitoring the changes of temperatures using a thermographic camera. As shown in Figure 2j‐k, we found all tested cell concentrations displayed notable temperature incasement, while there was no obvious temperature increased by the PBS. The photothermal conversion effect positively correlates with bacterial concentration. Notably, the changes of temperature (ΔT) increased by 16.8 °C and 17.8 °C and the temperature can reach 43.8 °C and 44.8 °C when the *R. sphaeroides* at the concentration of 7.2 × 10^9^ CFU mL^−1^ and 1.4 × 10^10^ CFU mL^−1^ (Figure 2j,k; Figure S4, Supporting Information). Thus, the photothermal conversion efficiency was similar at the concentration between 1.4 × 10^10^ and 7.2 × 10^9^ CFU mL^−1^, and we selected concentration of 7.2 × 10^9^ CFU/mL for the following experiments. As shown in Figure S5 (Supporting Information), the accelerated temperature increased with laser power intensity lower than 1.5 W cm^−2^, and temperature was similar under the power of 1.5 W cm^−2^ (52.3 °C) and 2.0 W cm^−2^ (53.5 °C). Therefore, the laser power of 1.5 W/cm^2^ was selected for photothermal experiments. Furthermore, following 4 repeated on‐and‐off cycles of 808 nm laser irradiation, *R. sphaeroides* shows no significant deterioration, highlighting its photothermal stability (Figure S6, Supporting Information). The photothermal conversion efficiency (*η*) of *R. sphaeroides* was calculated to be 7.4% (Figure S7, Supporting Information).^[^ ^21^ ^]^ Remarkably, *R. sphaeroides* displays a high degree of thermotolerance, proliferating even at 55 °C (Figure S8, Supporting Information), with no significant differences in proliferation under 808 nm laser irradiation compared to the conditions without laser irradiation. In contrast, for the bacteria without BChl, such as *Synechococcus elongatus*, *Bacillus thuringiensis*, and *Escherichia coli*, those stains showed no photothermal conversion ability upon exposure to 808 nm laser irradiation with the same power and cell concentration (Figure S9, Supporting Information), indicating the curial role of BChl in the photothermal conversion. Taking together, our results underscore the unique capabilities of *R. sphaeroides* in combining hydrogen production and photothermal effects under the control of NIR light, making it an effective candidate agent for cancer therapy.

### 2.2 In Vitro Hydrogen‐Photothermal Therapy

We first explore the efficiency of *R. sphaeroides* and BChl for producing H_2_ and heat simultaneously under the control of NIR light. As shown in Figure S10 (Supporting Information), the bacteria irradiated with 808 nm laser (1.5 W cm^−2^, the experimental group) could produce 42.2 µM H_2_ and increase the ΔT to 24.9 °C. While the control group, including the bacteria irradiated with 808 nm LED light (0.08 W cm^−2^), produces H_2_ (46.6 µM), which is comparable to the experimental group and represents hydrogen therapy. The BChl irradiated with 808 nm laser (1.5 W cm^−2^) only generates heat (ΔT = 23.0 °C) which represents photothermal therapy (PTT), and neither H_2_ nor heat was produced by the BChl irradiated with 808 nm LED light (0.08 W cm^−2^). Consequently, hydrogen therapy, PTT, and synergistic hydrogen‐photothermal therapy were implemented in vitro.

**Figure 3:**
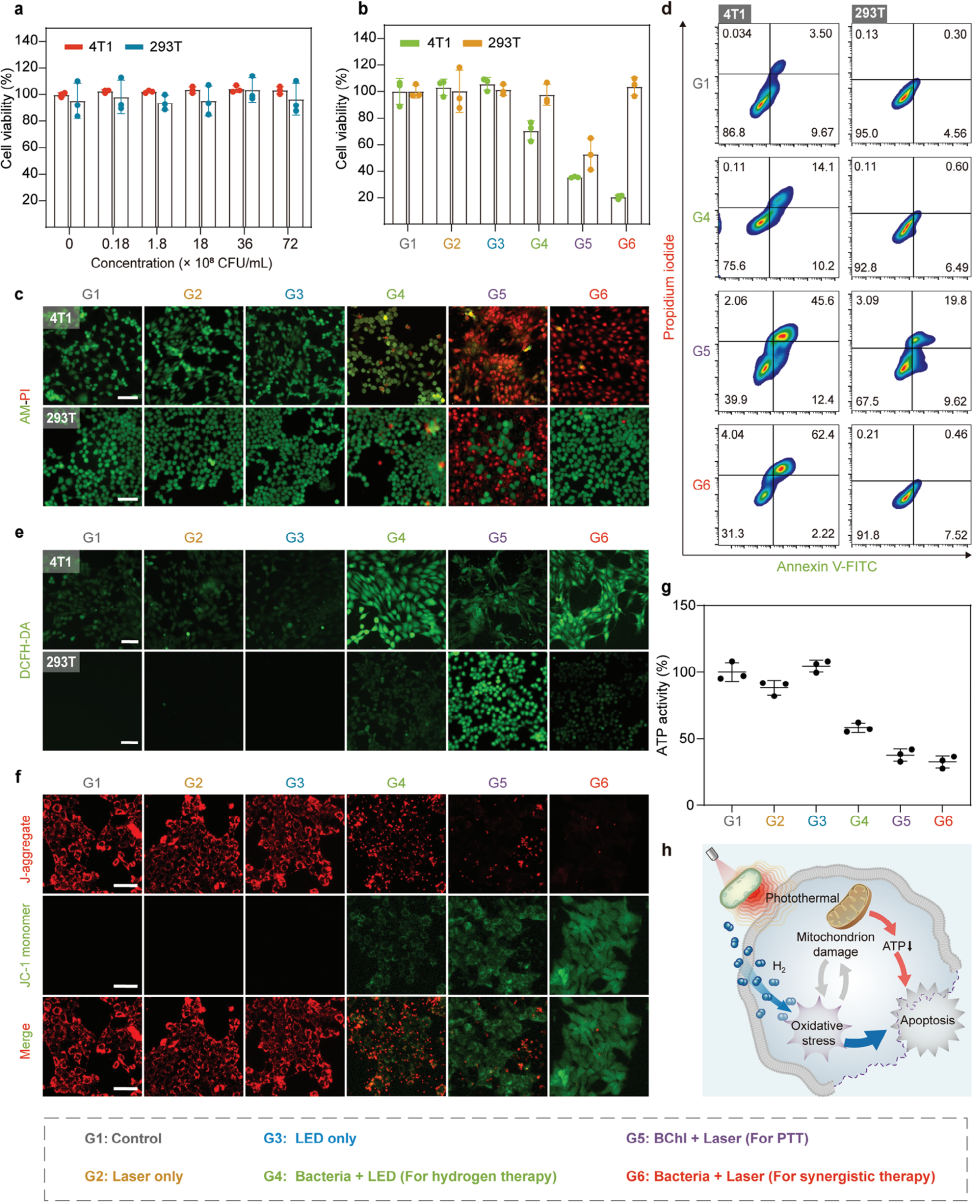
In vitro hydrogen‐photothermal therapy using *R. sphaeroides*. a) Cell viabilities of 4T1 cells and 293T cells incubated with *R. sphaeroides* at different concentrations (0, 0.18×10^8^, 1.8×10^8^, 18×10^8^, 36×10^8^, and 72×10^8^ CFU mL^−1^) (*n* = 3 biologically independent samples). b) Cell viabilities of 4T1 cells and 293T cells after different treatments (*n* = 3 biologically independent samples). c) Confocal microscopy images of 4T1 cells and 293T cells stained with Calcein‐AM and PI after different treatments (green: living cells; red: dead cells). Scale bars, 100 µm. d) Flow cytometry analysis of 4T1 cells and 293T cells stained with PI and Annexin V‐FITC. e) Confocal microscopy images of 4T1 cells and 293T cells stained with 2′,7′ – Dichlorofluorescein diacetate (DCFH‐DA) after different treatments. Scale bar, 100 µm. f) Confocal microscopy images of 4T1 cells stained with JC‐1 after different treatments. Scale bars, 50 µm. g) ATP activity in 4T1 cells after different treatments. (*n* = 3 biologically independent samples). h) Schematic illustration showing mechanism underlying *R. sphaeroides* mediated hydrogen‐photothermal therapy. Data are presented as mean values ± SD.

We evaluated the cytotoxicity of *R. sphaeroides* using Cell Counting Kit‐8 (CCK‐8) assay on a cancer cell line (4T1 cells) and a normal cell line (HEK‐293T cells). Without 808 nm laser/LED irradiation, *R. sphaeroides* exhibited negligible cytotoxicity, even at a concentration of 7.2×10^9^ CFU mL^−1^, demonstrating its biosafety in vitro (**Figure** **3**a). Hydrogen therapy (G4) using the bacteria with 808 nm LED light selectively killed cancer cells (Figure 3b). The BChl with 808 nm laser group, assigned for PTT (G5), was lethal to both cancer cells and normal cells. Notably, the bacteria with 808 nm laser irradiation (assigned hydrogen‐photothermal therapy, G6) demonstrates enhanced lethality to cancer cells compared to the PTT group, while minimizing damage to normal cells. Live/dead cell staining further confirmed that G6 induces the highest cell mortality among all groups in 4T1 cells, while maintain the activity of HEK‐293T cells, demonstrating the efficacy of the synergistic therapy (Figure 3c). In contrast, G5 led to cell death in HEK‐293T cells, indicating the potential of damage to normal cells caused by PTT. Flow cytometry following dual staining with fluorescein isothiocyanate (FITC)‐labeled Annexin V and propidium iodide (PI) revealed notably enhanced apoptosis in 4T1 cells at 64.6% for the G6 of hydrogen‐photothermal therapy, while only 8.0% apoptosis in HEK‐293T cells (Figure 3d). Those results indicated hydrogen‐photothermal therapy enhances anti‐cancer effects while minimizing damage to normal cells, indicated the selectivity and effectiveness of the anti‐cancer effects.

Intracellular ROS levels were assessed to evaluate redox homeostasis. 4T1 cells showed significant increases in ROS following hydrogen therapy and combined hydrogen‐photothermal therapy, indicating that H_2_ induces oxidative stress in cancer cells (Figure 3e). Conversely, HEK‐293T cells exhibited increased ROS production only after PTT with BChl and 808 nm laser irradiation. Notably, H_2_ helps restore normal cellular redox homeostasis, suggesting that the synergistic therapy may protect normal cells against oxidative damage caused by heat. Considering the naturally higher levels of ROS in cancer cells, hydrogen can initially lower the ROS levels because of its reducing properties. However, given the capacity for redox homeostasis in cells, this reduction might prompt a compensatory increase in ROS, potentially resulting in the death of cancer cells.^[^ ^10^, ^22^ ^]^ In contrast, H_2_ alleviates oxidative damage in normal cells induced by photothermal effect.^[^ ^3^, ^8^, ^23^ ^]^

We also investigated the impact of hydrogen‐photothermal therapy on mitochondrial membrane potential (MMP) and the intracellular ATP levels to elucidate the anti‐tumor mechanism. As shown in Figure 3f,g, hydrogen‐photothermal therapy caused a prominent decrease in MMP, and significant reductions in ATP levels were observed in 4T1 cells, primarily due to hyperthermia‐induced impairments. The relationship between excess ROS and mitochondrial damage is complementary. The excessive ROS leads to oxidative damage in mitochondria, which, when dysfunctional, releases substantial ROS in return.^[^ ^24^ ^]^ Thus, hydrogen therapy induces slight mitochondrial impairment in cancer cells due to excessive ROS generation, while PTT results in subtle ROS upregulation within cancer cells, possibly from the release of damaged mitochondria (Figure 3h). Furthermore, we delved into the effects of hydrogen‐photothermal therapy on other cellular organelles in cancer cells. The results indicated that this strategy induces endoplasmic reticulum (ER) stress and lysosomal damage in 4T1 cells (Figures S11 and S12, Supporting Information). These findings underscore the dual functionality of *R. sphaeroides* in combining hydrogen production and photothermal effects, offering a highly effective and selective approach for targeted cancer therapy.

### 2.3 Mechanism of Hydrogen‐Photothermal Therapy

**Figure 4:**
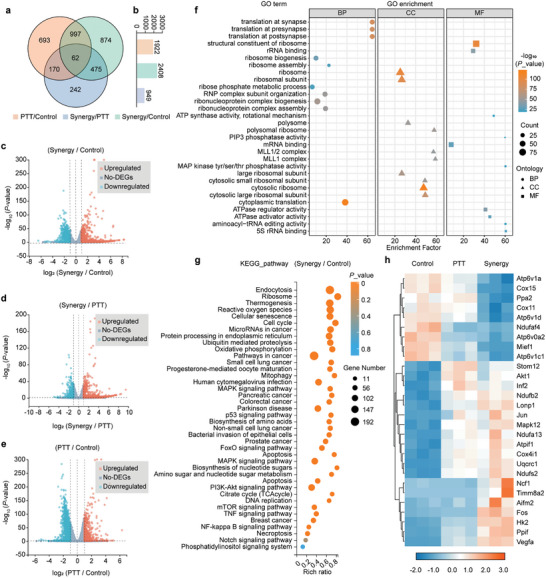
Transcriptional mechanism of *R. sphaeroides* mediated hydrogen‐photothermal therapy. a) Venn diagram of the transcriptomic profiles among control group, PTT group, and synergy therapy group. b) Different change genes statistics among control group, PTT group, and synergy therapy group. Volcano plots showing the identified upregulated and downregulated genes on 4T1 cells between c) synergy therapy group and control group, d) synergy therapy group and PTT group, e) PTT group and control group. f) GO pathway enrichment analysis of the identified DEGs between control group and synergy therapy group. g) KEGG pathway enrichment analysis of the identified DEGs between control group and synergy therapy group. h) Heat map illustrating the DEGs related to the mitochondrial respiratory chain, mitochondrial damage and ROS metabolic processes among control group, PTT group and synergy therapy group.

To elucidate the potential mechanisms underlying the anti‐tumor effects driven by *R. sphaeroides* in 4T1 cells, RNA sequencing (RNA‐seq) analysis was conducted. A total of 16083 expressed genes were identified (Figure S13, Supporting Information), with subsequent differential expression analysis revealing significant variances among different treatment groups (**Figure** **4**a,b; Figure S14, Supporting Information). Specifically, the hydrogen‐photothermal synergy therapy group exhibited 2408 differentially expressed genes (DEGs) compared to the control group (without treatment), with 979 genes up‐regulated and 1429 genes down‐regulated (| Fold change | ≥ 1, *p* < 0.05, Figure 4c). In contrast, only 949 DEGs were observed when compared with the PTT group alone (599 up‐regulated, 350 down‐regulated, Figure 4d). Furthermore, a comparison between the control group and PTT group revealed 1922 differential genes (623 up‐regulated, 1299 down‐regulated, Figure 4e), indicating that heat notably contributes to gene expression alteration in tumor cells within the context of synergistic therapy.

Gene Ontology (GO) pathway enrichment analysis and Kyoto Encyclopedia of Genes and Genomes (KEGG) analysis were performed on the DEGs to assess their involvement in metabolic pathways. The synergy therapy group displayed significant enrichment in pathways related to mitochondria function and signaling (Figure 4f). KEGG analysis further highlighted significant enrichment in pathways governing redox homoeostasis and mitochondria function, such as ROS production, oxidative phosphorylation, p53, mitogen‐activated protein kinase (MAPK) signaling pathway, and apoptosis (Figure 4g). The DEGs are enriched in the PI3K/Akt signaling pathway and the NF‐κB signaling pathway, suggesting that this synergistic strategy might induce cell apoptosis through these critical pathways.^[^ ^25^ ^]^ These findings suggest that the synergy therapy profoundly impacts redox homoeostasis and mitochondria function in tumor cells.

Analysis of DEGs within these pathways in heat maps revealed that genes such as cytochrome c oxidase assembly protein 15 (Cox15), associated with mitochondrial respiratory chain, were down‐regulated, while genes such as lon peptidase 1 (Lonp1), linked to mitochondrial damage, were up‐regulated after both PTT and synergistic therapy (Figure 4h). Thus, in this strategy, photothermal therapy might lead to mitochondrial dysfunction by aberrantly activating mitochondrial‐related signaling pathways or promoting atypical expression of certain mitochondrial proteins.^[^ ^26^ ^]^ Additionally, genes involved in ROS metabolic processes, such as neutrophil cytosolic factor 1 (Ncf1), were predominantly up‐regulated after synergistic therapy, suggesting that disruption of mitochondrial function is primarily attributed to heat, whereas alternations in ROS are mainly influenced by H_2_.^[^ ^27^ ^]^ Furthermore, up‐regulated genes such as growth arrest and DNA damage inducible alpha (Gadd45a), associated with stress signaling and injury response,^[^ ^28^ ^]^ and other significantly altered genes such as B‐cell lymphoma‐2 (Bcl‐2), associated with tumor suppression,^[^ ^29^ ^]^ indicated that the synergistic strategy might trigger the cell apoptosis process by regulating these downstream regulators (Figure S15, Supporting Information). The RNA‐seq results comprehensively demonstrate that *R. sphaeroides*‐mediated hydrogen‐photothermal therapy compromises mitochondrial function and disrupts redox homeostasis in cancer cells, leading to oxidative stress and ultimately resulting in apoptosis of tumor cells (Figure S16, Supporting Information).

### 2.4 In Vivo Hydrogen‐Photothermal Therapy

**Figure 5:**
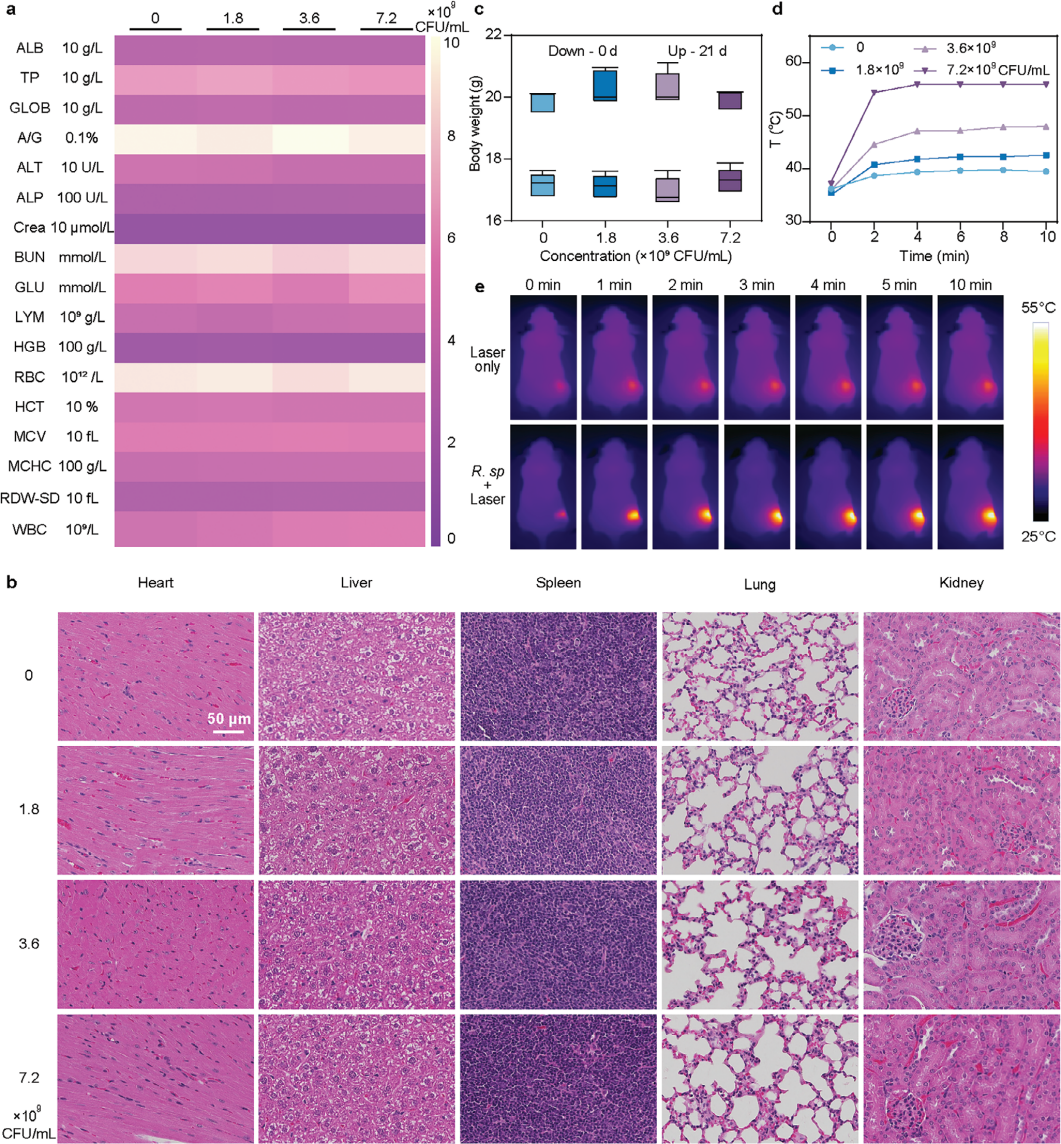
In vivo biocompatibility and photothermal performance investigation of *R. sphaeroides*. a) Blood routine and blood chemistry of the mice after treatment with *R. sphaeroides* at different concentrations (0, 1.8×10^9^, 3.6×10^9^, and 7.2×10^9^ CFU mL^−1^) (*n* = 5 biologically independent animals). b) Histological analyses of main organs from mice injected with *R. sphaeroides* at different concentrations (0, 1.8×10^9^, 3.6×10^9^ and 7.2×10^9^ CFU mL^−1^). c) The body weight changes of mice were observed over 21 days after they were intravenously injected with different concentrations (0, 1.8×10^9^, 3.6×10^9^, and 7.2×10^9^ CFU mL^−1^) of *R. sphaeroides* (*n* = 5 biologically independent animals). d) Photothermal temperature curves of 4T1 tumors region under 808 nm laser irradiation (1.5 W cm^−2^, 10 min) in untreated mice (Laser only) and *R. sphaeroides* injected mice (*R. sphaeroides* + Laser). e) Thermal images of 4T1 tumors under 808 nm laser irradiation (1.5 W cm^−2^, 10 min) in untreated mice and *R. sphaeroides* injected mice. Data are presented as mean values ± SD.

To evaluate the in vivo therapeutic efficacy of hydrogen‐photothermal treatment, we initially investigated the biocompatibility of *R. sphaeroides* by administering it intravenously to healthy BALB/c mice. Over a 21‐day observation period, no mortality occurred, and no significant changes in body weight were detected, indicating the absence of systemic toxicity from *R. sphaeroides* (**Figure** **5**c, and Table S1, Supporting Information). Furthermore, comprehensive blood routine and blood biochemistry assessed at the end of this period show that all indexes were within normal ranges, even at the concentration of 7.2×10^9^ CFU mL^−1^ (Figure 5a; Figure S17, Supporting Information), suggesting benign blood compatibility and no discernible toxicity to liver or kidney functions. Histopathological analyses via Hematoxylin and Eosin (H&E) staining of major organs including heart, liver, spleen, lung, and kidney, corroborated the high hemocompatibility, with no visible organ damage, confirming the compatibility of *R. sphaeroides* (Figure 5b). Consequently, the use of *R. sphaeroides* as a therapeutic agent has been shown to be safe in mice, yet more extensive safety evaluations on large animal models or humans are required for clinical application. Subsequently, 4T1 breast cancer tumor‐bearing mice model were constructed, and bacteria was peritumorally injected into the mice. During 808 nm laser irradiation at the tumor region, the temperature in the tumor region reached up to 55 °C for 10 min, which was sufficient to effectively ablate tumor cells (Figure 5d,e; Figure S18, Supporting Information), indicating the potential of *R. sphaeroides* for effective cancer cells ablation in vivo.

**Figure 6:**
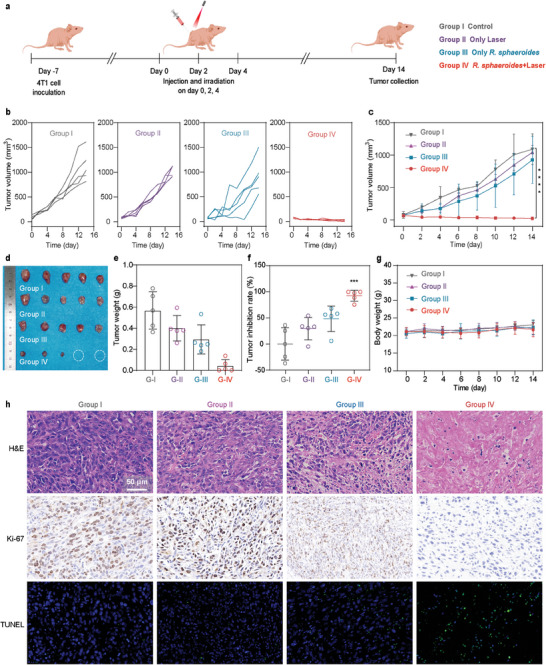
In vivo *R. sphaeroides* mediated hydrogen‐photothermal therapy. a) Schematic illustration showing the treatment schedule. b) Individual tumor growth curves on 4T1 tumor‐bearing mice after different treatments (*n* = 5 biologically independent animals). c) Average tumor growth curves on 4T1 tumor‐bearing mice after different treatments (*n* = 5 biologically independent animals). d) Photograph of the excised tumors from 4T1 tumor‐bearing mice after different treatments (*n* = 5 biologically independent animals). e) Tumor weight of the excised tumors from 4T1 tumor‐bearing mice after different treatments (*n* = 5 biologically independent animals). f) Tumor growth inhibition rate on 4T1 tumor‐bearing mice after different treatments (*n* = 5 biologically independent animals). g) The body weights of 4T1 tumor‐bearing mice after different treatments (*n* = 5 biologically independent animals). h) H&E, TUNEL and Ki‐67 staining assays of the tumor from 4T1 tumor‐bearing mice after different treatments. Data are presented as mean values ± SD. *P* values calculated by the Two‐tailed Student's *t* test (****P< 0.0001, ** *P*<0.01).

The anti‐tumor efficacy of hydrogen‐photothermal therapy was investigated in 4T1 tumor‐bearing mice, divided into four groups: control without any treatment (Group I), treated with laser irradiation (Group II), treated with *R. sphaeroides* (Group III), and treated with *R. sphaeroides* under laser irradiation (Group IV) (**Figure** **6**a). Notably, the Group IV showed significant tumor suppression (Figure 6b‐e), with tumor inhibition rate approaching 97% (Figure 6f). Furthermore, there were no obvious weight changes in all four treatment groups (Figure 6g), underscoring the biosafety of the therapeutic strategies. Mice were then euthanized, and the anti‐tumor activity was further studied by H&E staining, TdT‐mediated dUTP‐biotin nick end labeling (TUNEL) staining and Ki‐67 staining analysis (Figure 6h). Compared to other groups, H&E staining of tumor tissues showed extensive tumor tissue destruction, cellular and nuclear shrinkage, and complete nuclear disappearance in the Group IV, with no morphological changes in major organs (Figure 6h; Figure S19, Supporting Information). TUNEL staining images of tumor slices displayed a high proportion of apoptotic cells in the Group IV, while no or lower proportion in other groups. Additionally, the tumor proliferation and malignancy were significantly reduced in the Group IV compared with other control groups by Ki‐67 staining (Figure 6h). These findings collectively demonstrate the biocompatibility and remarkable efficacy of the synergistic hydrogen‐photothermal therapy using *R. sphaeroides*, promising significant advancements in cancer treatment.

## 3 Conclusion

This study introduces a distinct therapeutic modality that utilizes living photosynthetic bacteria, specifically *R. sphaeroides*, to concurrently generate H_2_ and induce hyperthermia under NIR light irradiation for targeted cancer therapy. The findings confirm that *R. sphaeroides* possesses strong NIR absorption capabilities, enabling sustained H_2_ production and efficient conversion of light energy into thermal energy directly at the tumor site. This dual functionality is critical for the therapeutic efficacy observed in the experiments. Both in vitro and in vivo results demonstrate that this hydrogen‐photothermal therapy approach yields selective and synergistic antitumor effects. Remarkably, the combination of H_2_ production with NIR‐triggered hyperthermia significantly enhances overall tumor suppression. This dual‐action strategy not only targets malignant cells with precision but also minimizes collateral damage to surrounding healthy tissues. The enhanced tumor suppression observed is attributed to the complementary effects of H_2_ and hyperthermia. H_2_ exerts its therapeutic effects by modulating ROS levels, leading to oxidative stress and apoptosis in cancer cells. Simultaneously, NIR‐induced hyperthermia disrupts mitochondrial function and further increases ROS levels, compounding the cytotoxic effects on tumor cells. Importantly, the synergistic therapy offers protection to normal cells and tissues, likely due to the bidirectional regulatory effects of H_2_. In normal cells, H_2_ enhances protective mechanisms, thereby mitigating oxidative damage and maintaining cellular homeostasis. In contrast, in tumor cells, H_2_ augments cytotoxicity through oxidative stress, demonstrating its selective action. This bidirectional regulation underscores the potential of H_2_ to differentiate between cancerous and healthy cells, making it a versatile therapeutic agent. Our study highlights the potential of hydrogen‐photothermal therapy as a comprehensive and effective strategy for cancer treatment, offering a promising alternative, especially for patients unresponsive to conventional treatments. The biocompatibility and high efficacy of *R. sphaeroides* in generating H_2_ and inducing hyperthermia suggest that this approach can be further developed and optimized for clinical applications. Future studies should focus on detailed investigations of the distribution, activity, and lifespan of *R. sphaeroides* in vivo to ensure biosafety and maximize therapeutic outcomes. In conclusion, this work establishes a representative approach utilizing living photosynthetic bacteria for hydrogen‐photothermal therapy, demonstrating significant advancements in targeted cancer treatment. In addition, many photosynthetic bacteria demonstrate remarkable photohydrogen production capabilities, which hold significant promise for hydrogen therapy. For instance, cyanobacteria, primitive prokaryotes, can perform photosynthesis and generate hydrogen under specific conditions. We anticipate that further studies will explore diverse photosynthetic bacteria in combination with other therapeutic modalities, aiming to facilitate the clinical translation of living therapeutics in the development of novel cancer treatment strategies.

## 4 Experimental Section

### Strains, Cells, and Materials

*R. sphaeroides* strain was obtained from the CAS Center for Excellence in Molecular Plant Sciences (CEMPS) and stored at −80 °C. The 4T1 murine breast cancer cell lines, and HEK‐293T embryonic kidney cells were sourced from Procell Life Science & Technology Co., Ltd (Wuhan, China). MB was purchased from Macklin (Shanghai, China). Roswell Park memorial institute‐1640 (RPMI 1640), Dulbecco's modified Eagle's medium (DMEM) and PBS were both obtained from VivaCell (Shanghai, China). Fetal bovine serum (FBS) and penicillin‐streptomycin were purchased from Gibco (Shanghai, China). CCK‐8 was obtained from Beyotime (Shanghai, China). Calcein‐AM and PI live/dead double stain kit was obtained from Solarbio (Beijing, China). DCFH‐DA assay kit, MMP assay kit with JC‐1, ATP assay kit, and Annexin V‐FITC apoptosis detection kit were purchased from Beyotime (Shanghai, China). The FastPure Cell/Tissue Total RNA Isolation Kit V2 was purchased from Vazyme (Nanjing, China). The H&E staining was purchased from Servicebio (Wuhan, China). The Ki‐67 polyclonal antibody was purchased from Proteintech (Wuhan, China). The one step TUNEL apoptosis assay kit was purchased from Beyotime (Shanghai, China).

### Bacterial Culture Condition

For bacterial culture, *R. sphaeroides* was streaked on LB solid plates and incubated at 37 °C for 24 h under sterile conditions. Colonies were selected and cultured overnight in LB liquid medium in a shaker at 37 °C and 220 rpm. The culture was then diluted 50‐fold into fresh medium and allowed to grow further. The bacteria were harvested by centrifugation at 4000 rpm for 5 min, and the pellet was resuspended in PBS for subsequent experiments.

### Characterization of R. Sphaeroides

SEM image of *R. sphaeroides* was acquired using a Phenom Pharos G2 FEG‐SEM (Eppendorf, Hamburg, Germany). Initially, the samples were fixed in 2.5% (v/v) glutaraldehyde in PBS (0.1 M, pH 7.0) overnight. Following fixation, the samples were washed three times with PBS (0.1 M, pH 7.0). The samples then underwent sequential dehydration with increasing concentrations of ethanol (30, 50, 70, 80, 90, and 95%) (v/v). Final dehydration was performed using a NP‐ZL3‐1K vacuum centrifugal concentrator (Gejian, Shanghai, China). Subsequently, the dehydrated samples were coated with gold‐palladium using an ISC 150 ion sputter (SuPro, Shenzhen, China) for 4 to 5 min and then observed under the Phenom Pharos G2 FEG‐SEM. UV‐Vis spectra were recorded using a GENESYS 150 spectrophotometer (Thermo Fisher, Waltham, USA). TEM images were captured using a HITACHI H‐7650 field emission transmission electron microscope at 120 Kv (Hitachi, Ltd, Japan).

### Extraction of the BChl

To extract the BChl from *R. sphaeroides*, 500 mL of fresh bacterial culture at the logarithmic (log) growth phase was collected and harvested by centrifugation at 8000 rpm for 5 min at 4 °C. Then, the pellet was washed with PBS for three times and resuspended in 20 mL of 60% (w/v) sucrose solution. The mixture was stirred for 24 h in the dark to extract the BChl. Finally, store the BChl at 4 °C in the dark.^[^ ^30^ ^]^

### Measurement of Hydrogen Production using MB Probe

Hydrogen production was measured using a MB probe. In the presence of hydrogen, MB is reduced to colorless leucomethylene blue (MBH_2_).^[^ ^31^ ^]^ In the presence of hydrogen, the blue color of MB is reduced to colorless leucomethylene blue (MBH_2_).^[^ ^31^ ^]^ The MB solution exhibits a characteristic absorption peak at 664 nm, and the release of hydrogen results in a decrease in this absorbance. Therefore, hydrogen release was measured based on the color change of the MB solution. Different concentrations of *R. sphaeroides* (0, 9.0×10^8^, 1.8×10^9^, 3.6×10^9^, 7.2×10^9^, and 1.4×10^10^ CFU mL^−1^) were dispersed in 4 mL of MB solution (9 µg mL^−1^). Subsequently, 1 µg mL^−1^ platinum (Pt) solution was added. The mixture was irradiated with NIR light (808 nm LED at 0.06 W cm^−2^ for 30 min). The absorption peak at 664 nm was detected using spectroscopy. The amount of hydrogen produced was calculated based on the decrease in the intensity of the absorbance peak and the standard curve of MB.

### Photothermal Performance Assessment

To evaluate the photothermal efficacy of *R. sphaeroides* at varying concentrations, bacterial suspensions were prepared in the following concentrations: 0, 9.0×10^8^, 1.8×10^9^, 3.6×10^9^, 7.2×10^9^, and 1.4×10^10^ CFU mL^−1^. 300 µL of each concentration was dispensed into separate wells of a 96‐well plate. Samples were exposed to an 808 nm laser (SLOC, Shanghai, China) with a power density of 1.0 W cm^−2^ for 5 min. PBS served as the negative control. Temperature measurements were recorded every second, and thermal images were captured at one‐minute intervals using a thermographic camera (Fotric, Shanghai, China). To assess the photothermal performance of *R. sphaeroides* under different power densities, 300 µL of bacterial suspension at a concentration of 7.2×10^9^ CFU mL^−1^ was irradiated for 5 min using an 808 nm laser at power densities of 0.5, 1.0, 1.5, or 2.0 W cm^−2^. Temperature changes were monitored using a Fotric 225S infrared thermal imager. To determine the impact of repeated irradiation on the photothermal efficiency of *R. sphaeroides*, bacterial suspension at a concentration of 7.2×10^9^ CFU mL^−1^ was exposed to an 808 nm laser at 1.5 W cm^−2^ for four cycles. Each cycle consisted of a 5‐minute exposure to 808 nm laser light at a power density of 1.5 W cm^−2^, followed by a 7‐minute cooling period. Temperature measurements were recorded every second using a thermographic camera to monitor the thermal response accurately. To evaluate the effects of 808 nm laser irradiation on bacterial viability, *R. sphaeroides* cultures were exposed to laser at a power density of 1.5 W cm^−2^ for 5 min. After irradiation, the viability was assessed by enumerating colony‐forming units (CFUs) on LB agar plates. Additionally, to examine the influence of hyperthermia on bacterial survival, cultures were subjected to various temperatures ranging from 30 to 60 °C for 10 min. Following thermal treatment, CFUs were quantified on LB agar plates to determine survival rates.

### Calculation of the Photothermal Conversion Efficiency

To assess the photothermal performance of the *R. sphaeroides* under an 808 nm laser, 300 µL of bacteria suspension was exposed to an 808 nm laser (1.5 W cm^−2^). Real‐time temperature changes were monitored using a thermographic camera with a precision of 1 s. The photothermal conversion efficiency (*η*) was calculated using Equation (1)

(1) $$\eta \;=\frac{h\cdot A\left[T_{Max\left(sample\right)}-T_{Sur\left(sample\right)}\right]-h\cdot A\left[T_{Max\left(water\right)}-T_{Sur\left(water\right)}\right]}{I\left(1-10^{-A_{808}}\right)}$$

where *T_max_* represents the maximum steady‐state temperature, *T_sur_* is the ambient temperature of the surrounding, *A* is the irradiated area, h is the heat transfer coefficient, *I* is the laser power, and *A*_808_ is the absorbance of *R. sphaeroides* at 808 nm.

### Cell Lines and Culture Conditions

4T1 murine breast cancer cells and HEK‐293T human embryonic kidney cells were cultured under specified conditions. The 4T1 cells were cultured in RPMI 1640 medium supplemented with 10% (v/v) foetal bovine serum (FBS) and 1% (v/v) antibiotics (penicillin‐streptomycin, 10 000 U mL^−1^). HEK‐293T cells were cultured in DMEM supplemented with 10% (v/v) FBS and 1% (v/v) antibiotics (penicillin‐streptomycin, 10 000 U mL^−1^). Both cell types were cultured in an incubator (Thermo Scientific) at 37 °C in an atmosphere of 5% (v/v) CO_2_ and 90% (v/v) relative humidity. For cell digestion and subculturing, 0.25% (w/v) trypsin was utilized.

### Cell Cytotoxicity Assay

The in vitro cytotoxicity of bacteria against 4T1 murine breast cancer cells and HEK‐293T human embryonic kidney cells was evaluated using the CCK‐8 assay. For the toxicity test, cells were seeded in a 96‐well plate at a density of 1×10^4^ cells per well. After a 24‐hour incubation, 100 µL of medium containing varying concentrations of bacteria was added. Following a further 4‐hour incubation, the supernatant in each well was discarded, and the cells were washed 3–5 times with PBS. Subsequently, 100 µL of medium mixed with 10 µL of CCK‐8 solution was added to each well. The plates were incubated for an additional 2–4 h before measuring the absorbance at 450 nm using a BioTek SYNERGY H1 microplate reader. The anti‐cancer effects of hydrogen‐photothermal therapy on 4T1 or HEK‐293T cells were evaluated using CCK‐8 assay. Cells were plated at a density of 1×10^4^ cells per well in a 96‐well plate. After a 24‐hour incubation, 100 µL of medium containing varying concentrations of bacteria was added. After a further 4‐hour incubation, different treatments were applied according to the designated groups: medium alone in darkness, an 808 nm laser at 1.5 W cm^−2^ for 10 min, an 808 nm LED at 0.08 W cm^−2^ for 10 min for the three control groups; bacteria (7.2×10^9^ CFU mL^−1^) and 808 nm LED (0.08 W cm^−2^, 10 min) irradiation for the hydrogen therapy group; BChl and 808 nm laser (1.5 W cm^−2^, 10 min) irradiation for the PTT group; and bacteria (7.2×10^9^ CFU mL^−1^) with 808 nm laser (1.5 W cm^−2^, 10 min) irradiation for the hydrogen‐photothermal therapy group. Following different treatments, the supernatant in each well was removed, and cells were washed 3–5 times with PBS, and 100 µL of medium with 10 µL CCK‐8 solution was added to each well. The plates were incubated for an additional 2–4 h before the absorbance at 450 nm was measured using a BioTek SYNERGY H1 microplate reader.

### Live/Dead Cell Staining Assay

The anti‐cancer effects of the therapies on 4T1 cells or HEK‐293T cells were further evaluated by a Calcein‐AM/PI dual staining assay. Cells were seeded into a 12‐well plate at a density of 1×10^5^ cells per well and cultured for 24 h, after which 1 mL of medium containing the experimental samples was added. Following another 4‐hour incubation, treatments were consistent with the aforementioned grouping. After different treatments, the supernatant in each well was discarded and cells were washed 3–5 times with PBS, then stained with Calcein‐AM and PI suspended in PBS. After a 30‐minute incubation at 37 °C, cells were imaged using an inverted fluorescence microscope (Nikon, ECLIPSE Ti2).

### Cellular ROS Assessment

The intracellular ROS levels in 4T1 and HEK‐293T cells were assessed using the DCFH‐DA fluorescent probe. Cells were seeded in a 12‐well plate at a density of 1×10^5^ cells per well. After culturing for 24 h, 1 mL of the sample‐containing medium was added to each well. Following an additional 4‐hour incubation, treatments corresponding to the previously described groupings were applied. After these treatments, the supernatant in each well was removed, and the cells were washed 3–5 times with PBS. Subsequently, the cells were stained with DCFH‐DA for 20 min. Fluorescence in the cells after different treatments was then examined using an inverted fluorescence microscope.

### Cell Apoptosis Measurement

The apoptosis of 4T1 or HEK‐293T cells was assessed using flow cytometry. Cells were seeded into a 12‐well plate at a density of 1×10^5^ cells per well and cultured for 24 h. After this period, 1 mL of medium containing the samples was added. Following an additional 4‐hour incubation, treatments corresponding to the previously described groupings were applied. After the treatments, the supernatant from each well was discarded and cells were washed 3–5 times with PBS. Subsequently, all cells were collected and stained with Annexin V‐FITC/PI for 15 min. The stained cells were then analyzed by flow cytometry to determine the percentage of apoptotic cells.

### Detection of Intracellular MMP and ATP Level

The levels of MMP in 4T1 cells were assessed using JC‐1 staining, which differentiate between monomeric form (green fluorescence) and aggregated form (red fluorescence). 4T1 cells were seeded into a 12‐well plate at a density of 1×10^5^ cells per well and cultured for 24 h. After this period, 1 mL of medium containing the samples in each well was added to each well. Following a further 4‐hour incubation, treatments corresponding to the previously described groupings were applied. After the treatments, the supernatant in each well was removed and cells were washed 3–5 times with PBS. The cells were stained with JC‐1 for 20 min, after which red and green fluorescence were detected using a fluorescence microscope. For the assay of intracellular ATP levels in 4T1 cells, the cells were lysed following the same treatment protocols. ATP levels were then measured using an ATP bioluminescent assay kit.

### Detection of ER Stress and Lysosomal Damage

The ER stress in 4T1 cells was assessed using the ER‐Tracker and the lysosomal damage in 4T1 cells was assessed using the Lyso‐Tracker. Cells were seeded in a 12‐well plate at a density of 1×10^5^ cells per well. After culturing for 24 h, 1 mL of the sample‐containing medium was added to each well. Following an additional 4‐hour incubation, treatments corresponding to the previously described groupings were applied. After these treatments, the supernatant in each well was removed, and the cells were washed 3–5 times with hank's balanced salt solution (HBSS). For the ER stress experiment, the cells were stained with ER‐Tracker for 15 min and then stained with Hoechst 33342 for 10 min. For the lysosomal damage experiment, the cells were stained with Lyso‐Tracker for 25 min. Fluorescence in the cells after different treatments was then examined using an inverted fluorescence microscope.

### In Vivo Biocompatibility of Photosynthetic Bacteria

In the animal experiments, the animal care and handling procedures adhered to the guideline of the Shenzhen Top Biotech Co.,Ltd Institutional Animal Care and Use Committee, IACUC (Approval number: TOP‐IACUC‐2022‐0158). Female BALB/c healthy mice (6 – 8 weeks old) were purchased from Zhuhai BesTest Bio‐Tech Co. Ltd. Mice were housed in ventilated cages under a 12‐hour light‐dark cycle (8:00 – 20:00 light; 20:00 – 8:00 dark), with constant room temperature (21 ± 1 °C) and relative humidity (40 – 70%). The mice were provided with food and water freely available at all times. To investigate the in vivo biocompatibility of photosynthetic bacteria, 20 healthy BALB/c mice were randomly divided into four groups. BALB/c healthy mice were sequentially intravenously injected with 150 µL of photosynthetic bacteria at varying doses (0, 1.8×10^9^, 3.6×10^9^, and 7.2×10^9^ CFU mL^−1^) dispersed in PBS. Mice injected with PBS served as the control group. Behavioral changes and body weights were monitored over a two‐week period. After two weeks, all mice were euthanized, and blood was collected via the orbital bleeding method for complete blood count (lymphocytes percentage, hemoglobin, red blood cells, hematocrit, means corpuscular volume, means corpuscular haemoglobin concentration, white blood cells, and red blood cell volume distribution width) and serum biochemistry analysis (albumin, total protein, globulin, albumin/globulin, alanine transaminase, alkaline phosphatase, creatinine, blood urea nitrogen, and blood glucose) using the blood analyzer BM830 (Baolingman, Beijing, China) and biochemical analyzer SMT‐120VP (Seamaty, Chengdu, China). The main organs including heart, liver, spleen, kidney, and lungs were preserved in a 10% (v/v) formalin solution and stained with H&E for histological analysis to evaluate the potential toxicity of the photosynthetic bacteria.

### Animal Model

Female BALB/c nude mice (6‐8 weeks old) were purchased from Zhuhai BesTest Bio‐Tech Co. Ltd. The husbandry conditions of mice were consistent with the previously described method. To establish the subcutaneous breast tumor model, 4T1 cells (1×10^7^ cells mL^−1^) suspended in 100 µL of PBS were subcutaneously injected into the right flank of each mouse. All animals were randomly assigned to experimental groups before any treatments. All experimental protocols were conducted in strict adherence to relevant ethical guidelines and regulations.

### Photothermal Performance of Photosynthetic Bacteria In Vivo

4T1 tumor‐bearing mice received peritumoral injection with 150 µL of photosynthetic bacteria suspension (7.2×10^9^ CFU mL^−1^) dispersed in PBS. Subsequently, the tumor areas were irradiated with an 808 nm laser at a power density of 1.5 W cm^−2^ for 10 min. Temperature changes and photothermal images during the treatment were recorded using a Fotric 225S infrared thermal imager.

### Anti‐Tumor Treatment In Vivo

When the volume of tumors reached ≈80 mm^3^, the 4T1 tumor‐bearing mice were randomly divided into four groups (*n* = 5) for different treatments: Group I, PBS; Group II, 808 nm laser (1.5 W cm^−2^, 10 min); Group III, photosynthetic bacteria (7.2×10^9^ CFU mL^−1^); Group IV, photosynthetic bacteria (7.2×10^9^ CFU mL^−1^) + 808 nm laser (1.5 W cm^−2^, 10 min). The mice from Group III and Group IV were peritumorally injected with 150 µL of bacteria on days 0, 2, and 5. The mice from Group I and Group II were peritumorally injected with 150 µL of PBS on days 0, 2, and 5. Laser irradiation was performed immediately following the injection. During the therapeutic period, the tumor volume and body weights of mice were monitored every other day. Tumor volume (TV) was calculated using Equation (2):

(2) $$\mathrm{TV}={\left(\mathrm{tumor}\;\mathrm{width}\right)}^{2}\times \left(\mathrm{tumor}\;\mathrm{length}\right)÷2$$

After 14 days of treatment, the mice were euthanized, and all tumors were collected. The tumor weights were measured, and the tumor inhibition rate (TIR) was calculated using Equation (3):

(3) $$\begin{array}{ccc} \mathrm{TIR} & = & \left(\mathrm{mean}\;\mathrm{tumor}\;\mathrm{weight}\;\mathrm{of}\;\mathrm{the}\;\mathrm{control}\;\mathrm{group}\right.\\ & & \left.-\mathrm{mean}\;\mathrm{tumor}\;\mathrm{weight}\;\mathrm{of}\;\mathrm{the}\;\mathrm{treatment}\;\mathrm{group}\right)/\\ & & \mathrm{mean}\;\mathrm{tumor}\;\mathrm{weight}\;\mathrm{of}\;\mathrm{control}\;\mathrm{group}\;\;\times \;\;100\% \end{array}$$

### Histological Analysis

On day 3 of therapy, one mouse from each group was randomly selected and euthanized. The major organs (heart, spleen, lungs, liver, and kidneys) and tumor tissues were harvested and immediately fixed in 10% (v/v) neutral buffered formalin solution. Tissue sections were prepared and subsequently stained with H&E for histological evaluation. Immunohistochemical staining was performed by the Ki‐67 polyclonal antibody to assess the expression of the proliferation marker Ki‐67. Additionally, a TUNEL immunofluorescent assay was conducted by the one step TUNEL apoptosis assay kit to detect apoptotic cells in the tissue sections.

### Biosafety of Hydrogen‐Photothermal Therapy In Vivo

The body weights of mice and the H&E‐stained histology of the main organs were used to assess the biosafety of hydrogen‐photothermal therapy in vivo. During the therapy, the body weights of mice were recorded every other day. For histological assessment via H&E‐stained, major organs including the heart, liver, spleen, lung, and kidney were excised from mice after a 14‐day treatment. The harvested tissues were immersion‐fixed in 4% (v/v) paraformaldehyde solution and subsequently paraffin‐embedded for sectioning and H&E staining procedures.

### Statistical Analysis

All results in this study were recorded as means ± SD. Statistical analysis was performed by the Two‐tailed Student's *t* test to identify significant differences. Differences were considered significant when **P < 0.01, ***P < 0.001, and ****P < 0.0001. All data were analyzed by and Graphpad Prism 8.0.

## Conflict of Interest

The authors declare no conflict of interest.

## Supporting information



Supporting Information

## Data Availability

The data that support the findings of this study are available from the corresponding author upon reasonable request.

## References

[1] a) F. Bray , M. Laversanne , H. Sung , J. Ferlay , R. L. Siegel , I. Soerjomataram , A. Jemal , CA. Cancer J. Clin. 2024, 74, 229; 38572751 10.3322/caac.21834

[2] a) Z. Cheng , M. Li , R. Dey , Y. Chen , J. Hematol. Oncol. 2021, 14, 85; 34059100 10.1186/s13045-021-01096-0 PMC8165984

[3] a) Z. Jin , L. Jiang , Q. He , Nat. Commun. 2024, 15, 3857; 38719843 10.1038/s41467-024-48319-9 PMC11079063

[4] a) Y. Liang , W. Ruan , Y. Jiang , R. Smalling , X. Yuan , H. K. Eltzschig , Nat. Rev. Cardiol. 2023, 20, 723; 37308571 10.1038/s41569-023-00886-y PMC11014460

[5] a) M. Carlstrom , Nat. Rev. Nephrol. 2021, 17, 575; 34075241 10.1038/s41581-021-00429-z PMC8169406

[6] a) J. L. Wallace , R. Wang , Nat. Rev. Drug Discov. 2015, 14, 329; 25849904 10.1038/nrd4433

[7] a) J. D. Byrne , D. Gallo , H. Boyce , S. L. Becker , K. M. Kezar , A. T. Cotoia , V. R. Feig , A. Lopes , E. Csizmadia , M. S. Longhi , J. S. Lee , H. Kim , A. J. Wentworth , S. Shankar , G. R. Lee , J. Bi , E. Witt , K. Ishida , A. Hayward , J. L. P. Kuosmanen , J. Jenkins , J. Wainer , A. Aragon , K. Wong , C. Steiger , W. R. Jeck , D. E. Bosch , M. C. Coleman , D. R. Spitz , M. Tift , et al., Sci. Transl. Med. 2022, 14, eabl4135; 35767653 10.1126/scitranslmed.abl4135 PMC9576196

[8] a) Y. Htun , S. Nakamura , T. Kusaka , Pediatr. Res. 2021, 89, 753; 32505123 10.1038/s41390-020-0998-z

[9] M. Dole , F. R. Wilson , W. P. Fife , Science 1975, 190, 152. 1166304 10.1126/science.1166304

[10] a) E. C. Cheung , K. H. Vousden , Nat. Rev. Cancer 2022, 22, 280; 35102280 10.1038/s41568-021-00435-0

[11] Y. Wu , M. Yuan , J. Song , X. Chen , H. Yang , ACS Nano 2019, 13, 8505. 31329427 10.1021/acsnano.9b05124

[12] a) B. Zhao , Y. Wang , X. Yao , D. Chen , M. Fan , Z. Jin , Q. He , Nat. Commun. 2021, 12, 1345; 33649319 10.1038/s41467-021-21618-1 PMC7921091

[13] a) M. N. Z. Mohd Noor , A. S. Alauddin , Y. H. Wong , C. Y. Looi , E. H. Wong , P. Madhavan , C. H. Yeong , Asian Pac. J. Cancer Prev. 2023, 24, 37; 36708550 10.31557/APJCP.2023.24.1.37 PMC10152878

[14] a) G. Suresh , P. Kumari , S. Venkata Mohan , Bioresour. Technol. 2023, 380, 129007; 37061171 10.1016/j.biortech.2023.129007

[15] a) H. Yan , M. Fan , H. Liu , T. Xiao , D. Han , R. Che , W. Zhang , X. Zhou , J. Wang , C. Zhang , X. Yang , J. Zhang , Z. Li , J Nanobiotechnol. 2022, 20, 280; 10.1186/s12951-022-01440-7 PMC9199139 35705974

[16] E. Eroglu , A. Melis , Bioresour. Technol. 2011, 102, 8403. 21463932 10.1016/j.biortech.2011.03.026

[17] V. W. Kelly , B. K. Liang , S. J. Sirk , ACS Synth. Biol. 2020, 9, 3184. 33205966 10.1021/acssynbio.0c00444

[18] a) S. L. Porter , G. H. Wadhams , J. P. Armitage , Trends Microbiol. 2008, 16, 251; 18440816 10.1016/j.tim.2008.02.006

[19] N. Basak , A. K. Jana , D. Das , D. Saikia , Int. J. Hydrogen Energ. 2014, 39, 6853.

[20] L. Gabrielyan , H. Sargsyan , A. Trchounian , Microb. Cell Fact. 2015, 14, 131. 26337489 10.1186/s12934-015-0324-3 PMC4558839

[21] W. Zhen , Y. Liu , L. Lin , J. Bai , X. Jia , H. Tian , X. Jiang , Angew. Chem. Int. Ed. Engl. 2018, 57, 10309. 29888846 10.1002/anie.201804466

[22] D. Trachootham , J. Alexandre , P. Huang , Nat. Rev. Drug Discov. 2009, 8, 579. 19478820 10.1038/nrd2803

[23] a) Y. He , B. Zhang , Y. Chen , Q. Jin , J. Wu , F. Yan , H. Zheng , ACS Appl. Mater. Interfaces 2017, 9, 21190; 28557412 10.1021/acsami.7b05346

[24] a) J. Zhang , C. M. Simpson , J. Berner , H. B. Chong , J. Fang , Z. Ordulu , T. Weiss‐Sadan , A. P. Possemato , S. Harry , M. Takahashi , T. Y. Yang , M. Richter , H. Patel , A. E. Smith , A. D. Carlin , A. F. Hubertus de Groot , K. Wolf , L. Shi , T. Y. Wei , B. R. Durr , N. J. Chen , T. Vornbaumen , N. O. Wichmann , M. S. Mahamdeh , V. Pooladanda , Y. Matoba , S. Kumar , E. Kim , S. Bouberhan , E. Oliva , et al., Cell 2023, 186, 2361; 37192619 10.1016/j.cell.2023.04.026 PMC10225361

[25] a) L. Yang , P. Shi , G. Zhao , J. Xu , W. Peng , J. Zhang , G. Zhang , X. Wang , Z. Dong , F. Chen , H. Cui , Signal Transduct. Target. Ther. 2020, 5, 8. 32296030 10.1038/s41392-020-0110-5 PMC7005297

[26] a) S. Dennerlein , P. Rehling , R. Richter‐Dennerlein , FEBS Lett. 2023, 597, 1569; 37247261 10.1002/1873-3468.14671

[27] R. Holmdahl , O. Sareila , L. M. Olsson , L. Backdahl , K. Wing , Immunol. Rev. 2016, 269, 228. 26683156 10.1111/imr.12378

[28] X. Palomer , J. M. Salvador , C. Grinan‐Ferre , E. Barroso , M. Pallas , M. Vazquez‐Carrera , Med. Res. Rev. 2024, 44, 1375. 38264852 10.1002/med.22015

[29] A. Basu , Pharmacol. Ther. 2022, 230, 107943. 34182005 10.1016/j.pharmthera.2021.107943

[30] M. Ramos‐Morales , M. Aguirre‐García , O. Cortés‐Zavaleta , H. Ruiz‐Espinosa , K. H. Estévez‐Sánchez , C. E. Ochoa‐Velasco , I. I. Ruiz‐López , Food Bioprod. Process. 2024, 144, 266254529.

[31] T. Seo , R. Kurokawa , B. Sato , Med. Gas Res. 2012, 2, 22273079. 10.1186/2045-9912-2-1 PMC3309943 22273079

