# Supplementary material for: Living Therapeutics for Synergistic Hydrogen‐Photothermal Cancer Treatment by Photosynthetic Bacteria
Source: Adv Sci (Weinh). 2024 Nov 4;12(1):2408807. doi: 10.1002/advs.202408807 (PMC11714200; doi:10.1002/advs.202408807)
Supplement: Supplementary file 1 — Supporting Information [file ADVS-12-2408807-s001.docx]

Supporting Information

**Living Therapeutics for Synergistic Hydrogen-Photothermal Cancer Treatment by Photosynthetic Bacteria**

*Yingyi Zhang, Xiaolian Deng, Lili Xia, Jianghui Liang, Meng Chen, Xiaoling Xu, Wei Chen, Jianwei Ding, Chengjie Yu, Limei Liu, Yang Xiang, Yiliang Lin, Fangfang Duan*, Wei Feng*, Yu Chen*, Xiang Gao**

*Correspondence author. Email: [chenyuedu@shu.edu.cn](mailto:chenyuedu@shu.edu.cn) (C. Y.), [fengw@shu.edu.cn](mailto:fengw@shu.edu.cn) (W. F.), [duanff3@mail.sysu.edu.cn](mailto:duanff3@mail.sysu.edu.cn) (F. D.), [gaoxiang@siat.ac.cn](mailto:gaoxiang@siat.ac.cn) (X. G.)


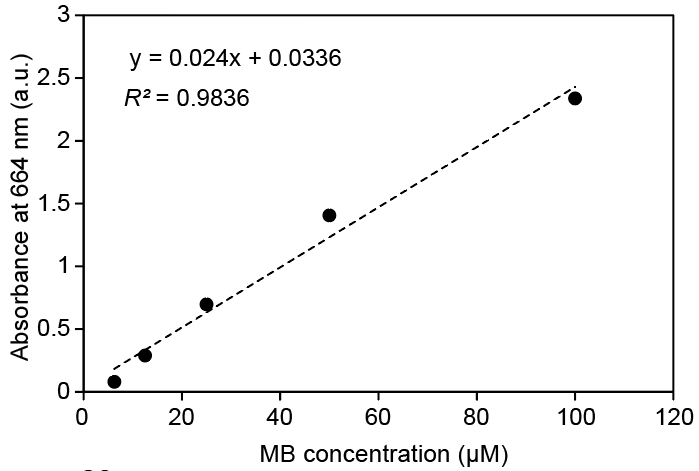


**Figure S1.** The linearly fitted standard curve of absorption intensity *vs* MB concentration, which was measured using the UV-Vis spectroscopy.


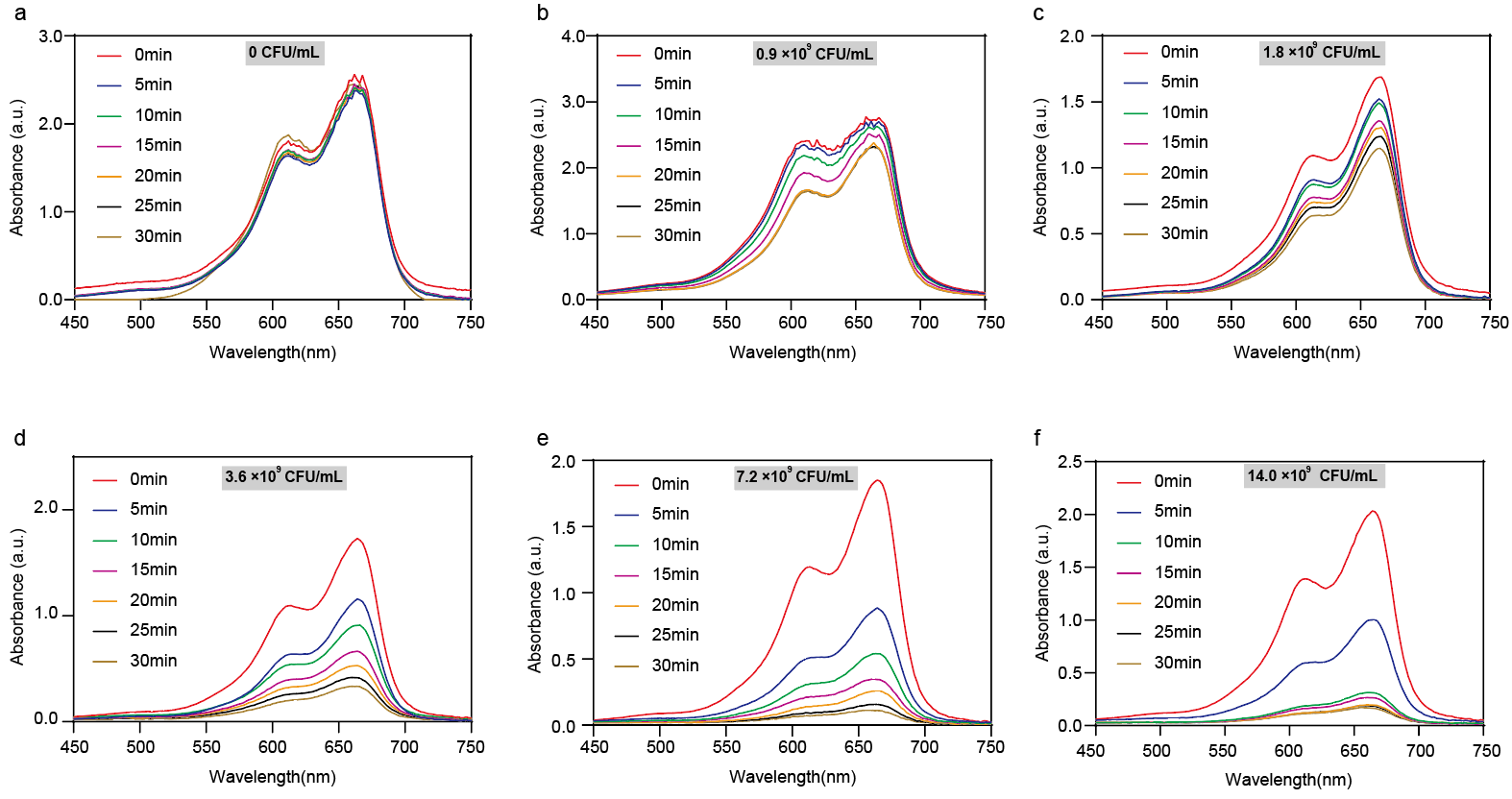


**Figure S2.** UV-Vis absorbance spectra of MB solution illustrating hydrogen production by photosynthetic bacteria at different concentrations (0, 0.9×10^9^, 1.8×10^9^, 3.6×10^9^, 7.2×10^9^, and 14×10^9^ CFU/mL) (a-f) suspended in PBS.

**
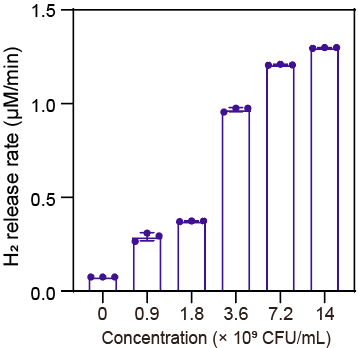
**

**Figure S3.** H_2_ release rate of photosynthetic bacteria at different concentrations (0, 0.9×10^9^, 1.8×10^9^, 3.6×10^9^, 7.2×10^9^, and 14×10^9^ CFU/mL) under NIR irradiation at the 30 min.

**
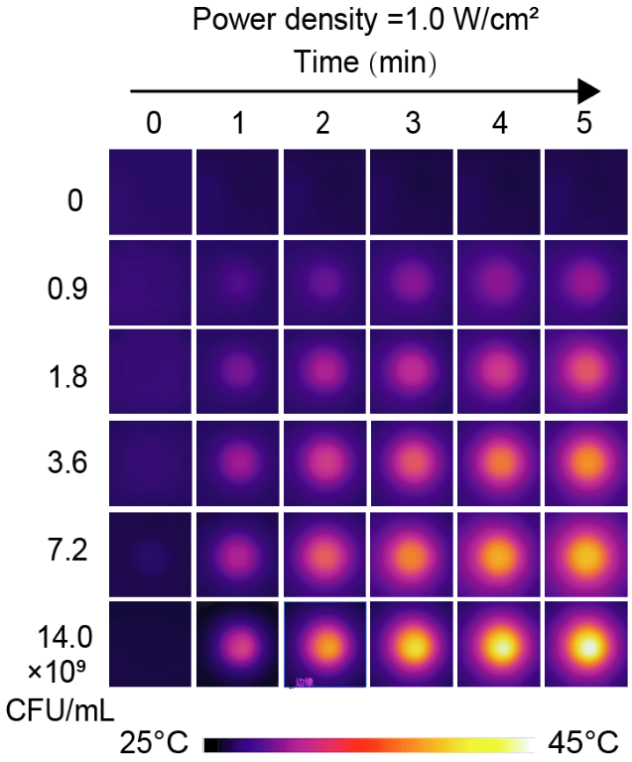
**

**Figure S4.** Infrared thermal images of bacteria at different concentrations (0, 0.9×10^9^, 1.8×10^9^, 3.6×10^9^, 7.2×10^9^, and 14×10^9^ CFU/mL) under exposure to an 808 nm laser (1.0 W/cm^2^).


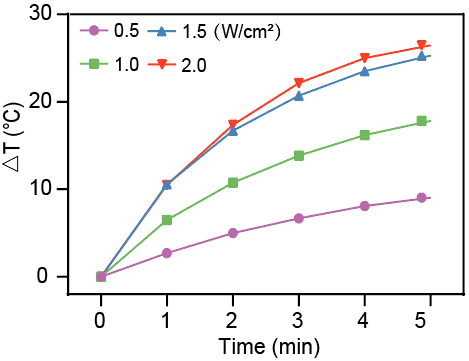


**Figure S5.** The temperature curves of bacteria (7.2 × 10^9^ CFU/mL) suspended in PBS under various power intensities of irradiation (0.5, 1.0, 1.5, 2.0 W/cm^2^). The graph shows the temperature change (ΔT) as a function of time for each power density.


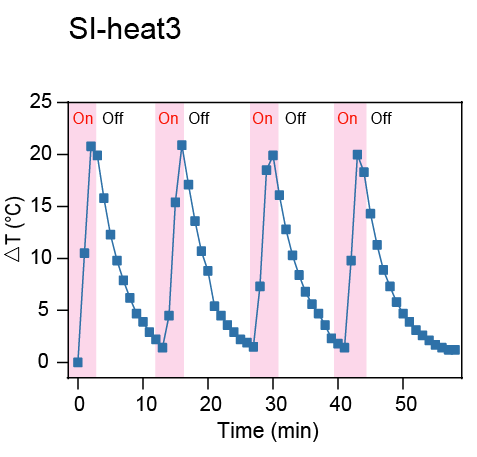


**Figure S6.** The cyclic photothermal tests of bacteria for four irradiation/cooling cycles (7.2 × 10^9^ CFU/mL, power density = 1.5 W/cm²). The ΔT is shown as a function of time, with periods of laser irradiation marked as 'On' and cooling periods marked as 'Off'.


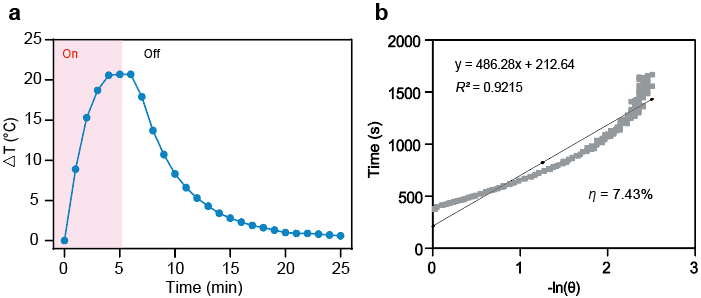


**Figure S7.** Calculation of photothermal conversion efficiency in photosynthetic bacteria. (a) Photothermal conversion characterization of the bacteria at the concentration of 7.2×10^9^ CFU/mL by an 808 nm laser at the power density of 1.5 W/cm^2^. (b) Time constant for heat transfer from the system τs, calculated by using the data after cooling period.


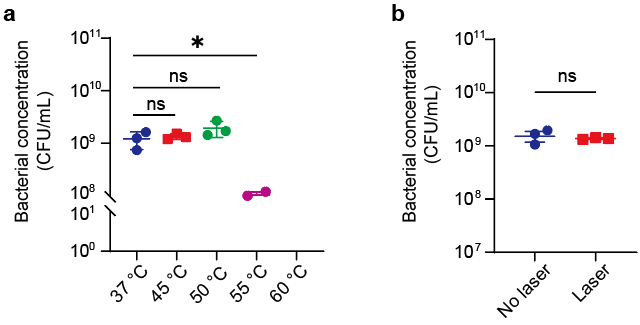


**Figure S8.** Detection of the thermal tolerance and laser tolerance of photosynthetic bacteria. (a) Colony counting of the bacteria treated thermally at different temperatures (37℃, 45℃, 50℃, 55℃, 60℃) and then re-cultured for 24 hours at 37℃. (b) Colony counting of the bacteria which was treated with or without irradiation of 808 nm laser (1.5 W/cm^2^, 10 min) and then re-cultured for 24 hours at 37℃. *P* values calculated by the Two-tailed Student’s *t* test (* *P* ≤ 0.05).


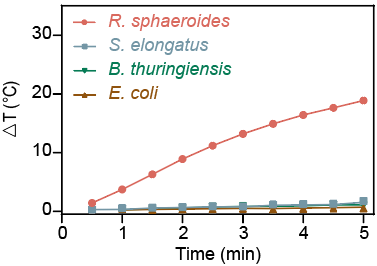


**Figure S9.** Photothermal effect of different bacterial strains. The graph shows the ΔT as a function of time for four bacterial strains at the same concentration: *Rhodobacter sphaeroides*, *Synechococcus elongatus*, *Bacillus thuringiensis*, and *Escherichia coli* under irradiation of 808 nm laser (1.0 W/cm^2^, 5 min).


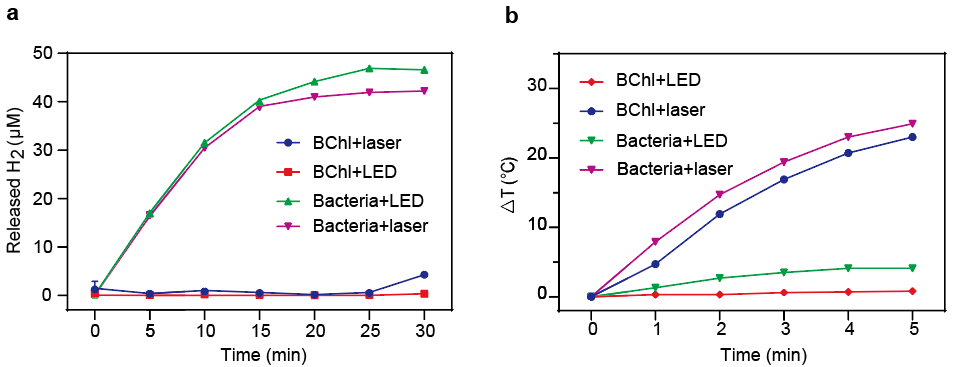


**Figure S10.** Detection of the H_2_ release and photothermal performance in photosynthetic bacteria and BChl. (a) H_2_ released in different treatments of bacteria (7.2×10^9^ CFU/mL) or BChl under irradiation of laser or LED. (b) Photothermal temperature curves of bacteria (7.2×10^9^ CFU/mL) or BChl under irradiation of laser or LED. Laser parameter: 808 nm, 1.5 W/cm^2^. LED parameter: 808 nm, 0.08 W/cm^2^.


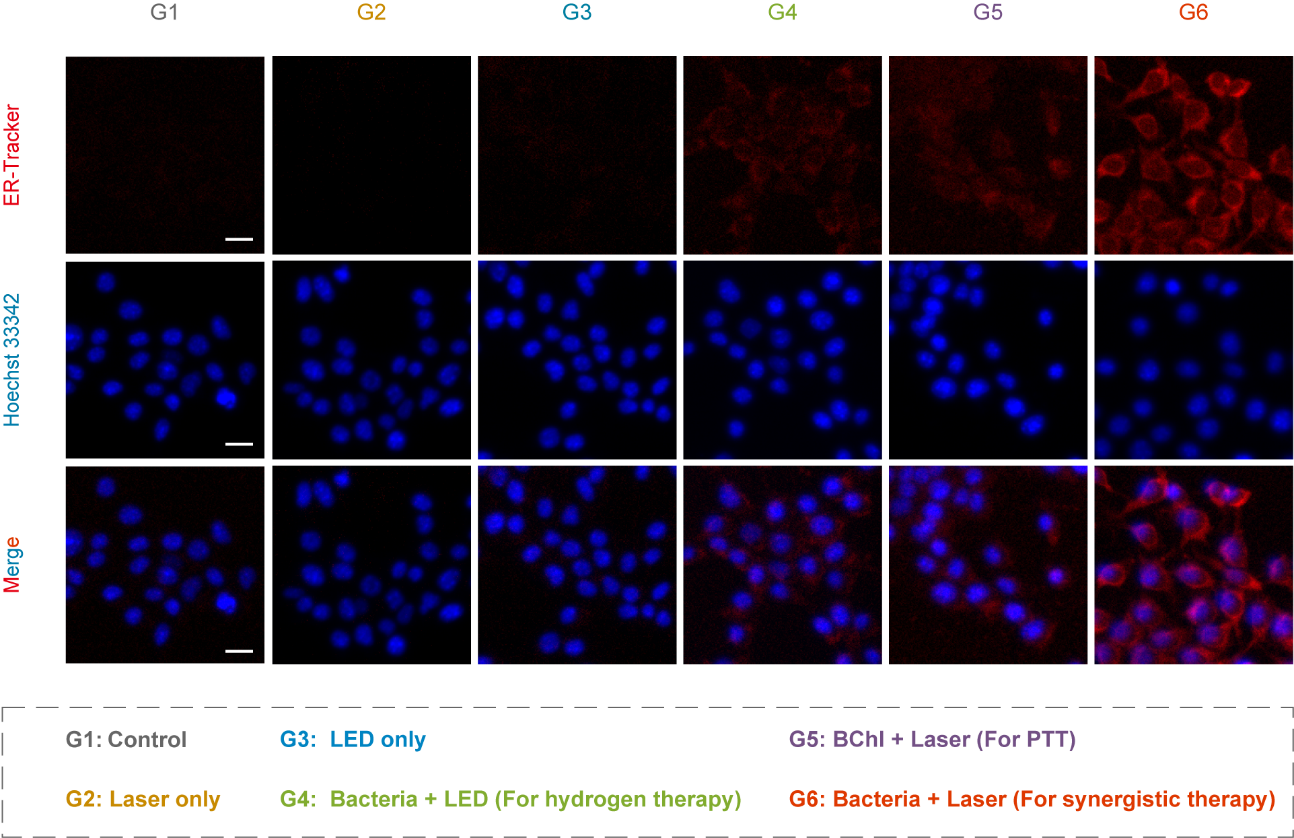


**Figure S11.** Confocal microscopy images of 4T1 cells stained with ER-Tracker and Hoechst 33342 after different treatments. Scale bars, 20 μm.


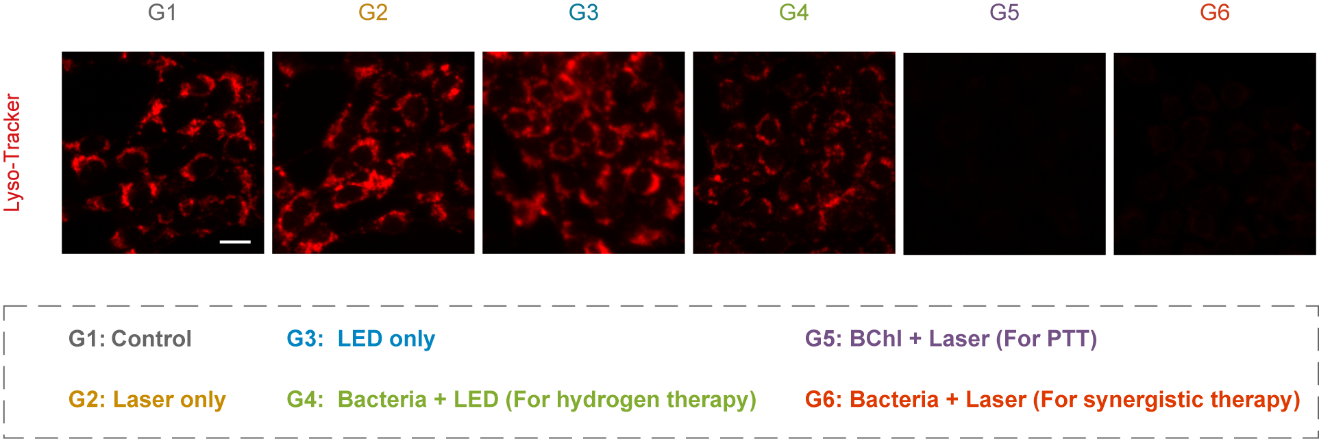


**Figure S12.** Confocal microscopy images of 4T1 cells stained with Lyso-Tracker after different treatments. Scale bars, 20 μm.


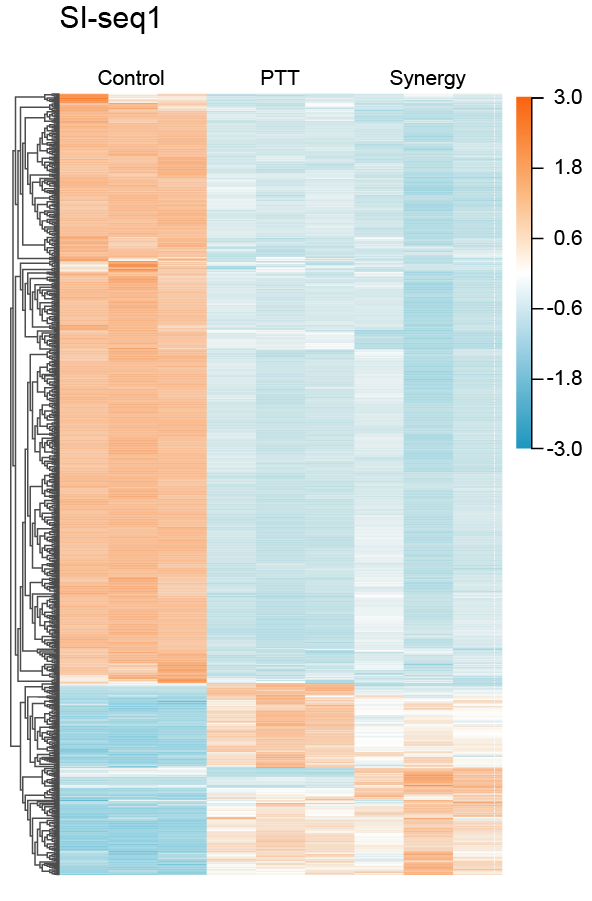


**Figure S13.** WPGMA-weighted hierarchical clustering of gene expression levels in RNA samples retrieved from 4T1 cells after different treatments. The heatmap displays the gene expression levels under three conditions: Control, photothermal therapy (PTT), and synergy therapy (Synergy). The color scale represents the level of gene expression, with orange indicating upregulation and blue indicating downregulation.


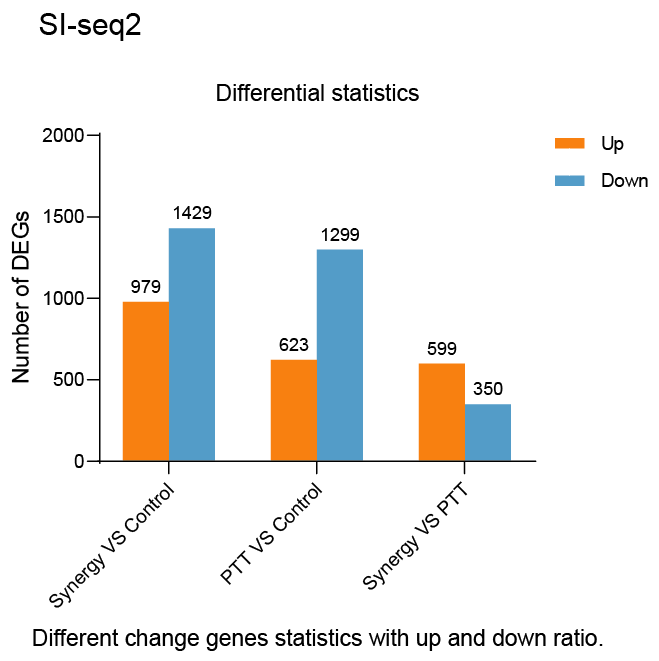


**Figure S14.** Differential gene change statistics with up and down ratios. The bar graph shows the number of differentially expressed genes (DEGs) that are upregulated (orange) and downregulated (blue) under three comparisons: Synergy vs. Control, PTT vs. Control, and Synergy vs. PTT.


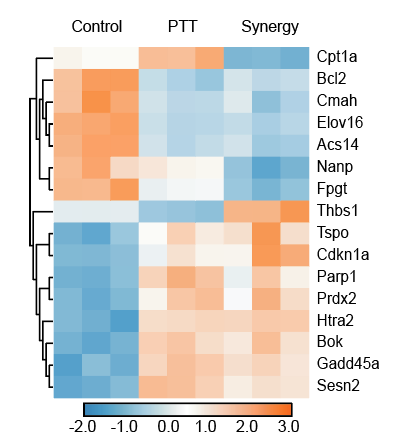


**Figure S15.** Heat map illustrating the DEGs between the control group, PTT group, and synergy therapy group. Each row represents a different gene, and each column represents a sample from one of the groups. The color scale ranges from blue (low expression) to orange (high expression), indicating the level of gene expression. Genes such as Cpt1a, Bcl2, Cmah, Elov16, Acs14, Nanp and Fpgt are involved in cell metabolism, Genes such as Thbs1, Tspo, Cdkn1a, Parp1, Prdx2, Htra2, Box, Gadd45a and Sesn2 are involved in tumor suppression.


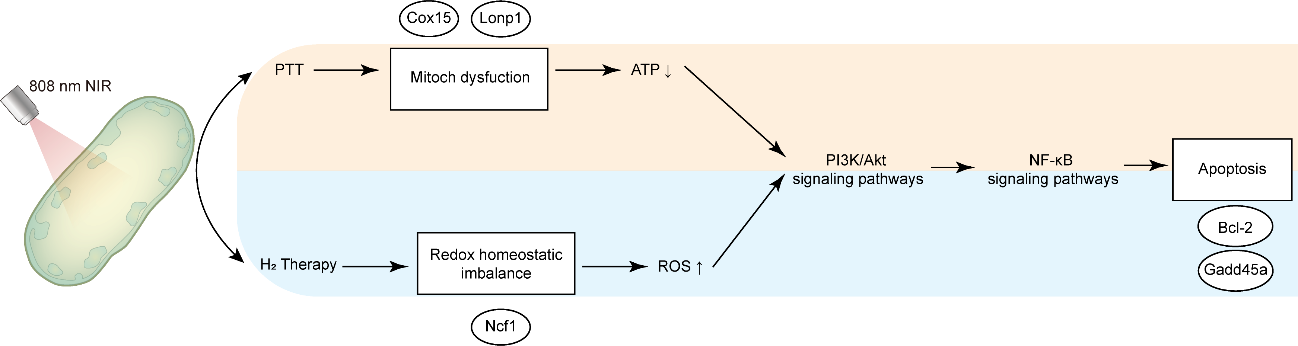


**Figure S16**. The detailed scheme to illustrate the pathways of the underlying biological mechanism. Cox15, Cytochrome c oxidase 15; Lonp1, lon peptidase 1; Ncf1, Neutrophil cytoplasmic factor 1; Bcl-2, B-cell lymphoma-2; Gadd45a, growth arrest and DNA damage inducible alpha.


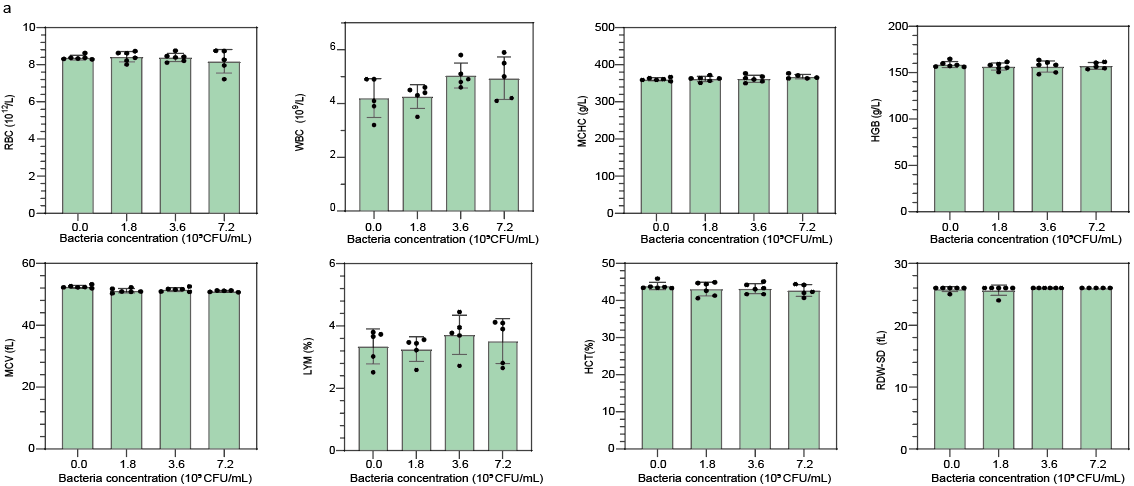


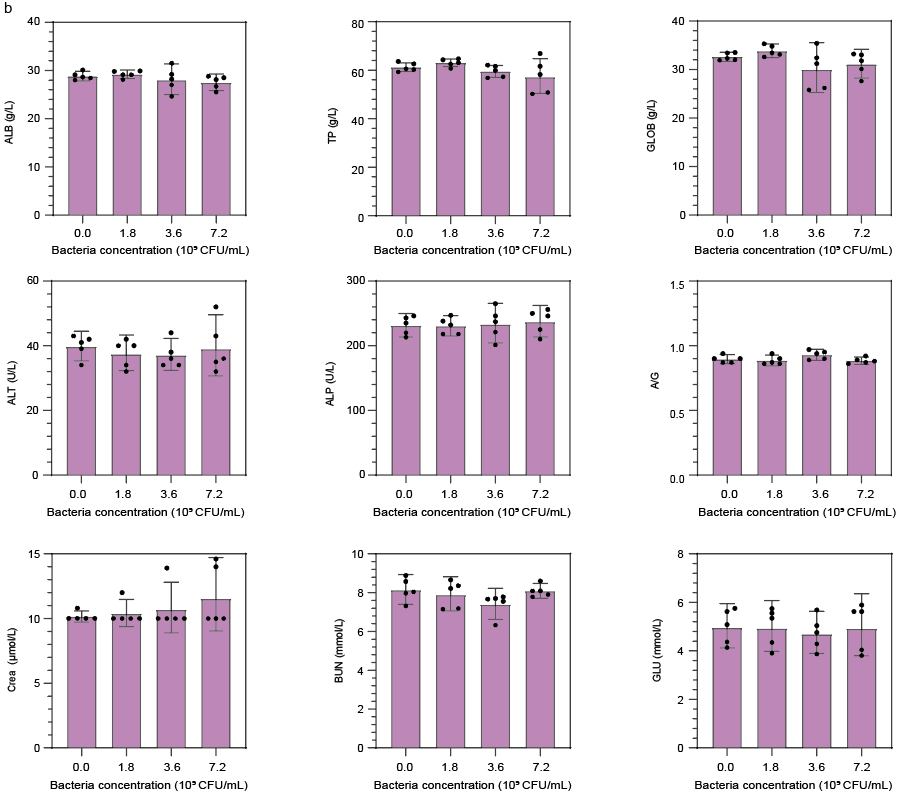


**Figure S17.** Blood routine and blood chemistry data of healthy female BALB/c mice intravenously injected with *R. sphaeroides* at different doses of 0, 1.8 × 10^9^, 3.6 × 10^9^ and 7.2 × 10^9^ CFU/mL per mouse at 1-day post-injection. (a) Blood routine parameters include red blood cells (RBC), means corpuscular volume (MCV), white blood cells (WBC), lymphocytes percentage (LYM), means corpuscular haemoglobin concentration (MCHC), hemoglobin (HGB), hematocrit (HCT), and red blood cell volume distribution width (RDW-SD) levels in the blood of mice injected with different doses of bacteria. (b) Blood chemistry parameters include albumin (ALB), total protein (TP), globulin (GLOB), albumin/globulin (A/G), alanine transaminase (ALT), alkaline phosphatase (ALP), creatinine (Crea), blood urea nitrogen (BUN), and blood glucose (GLU) in mice injected with different doses of bacteria. Statistic was based on five mice per data point.


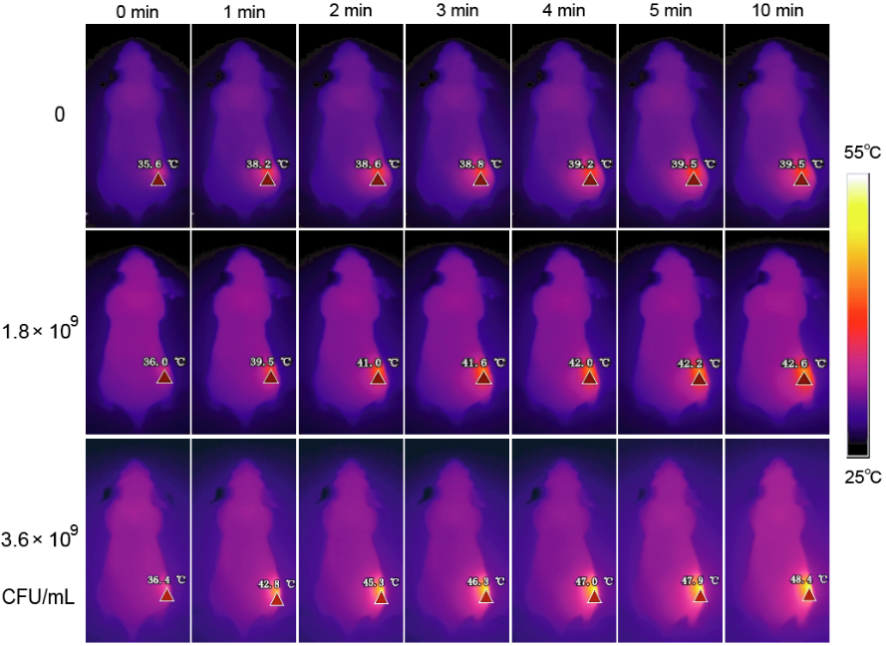


**Figure S18.** Photothermal images of 4T1 tumor-bearing mice in the only laser group (periatumorally injected with PBS) and laser + *R. sphaeroides* group (periatumorally injected with 1.8×10^9^ CFU/mL and 3.6×10^9^ CFU/mL of the bacteria) during 10 minutes of irradiation with an 808 nm (1.5 W/cm^2^) laser at the tumor region.

**
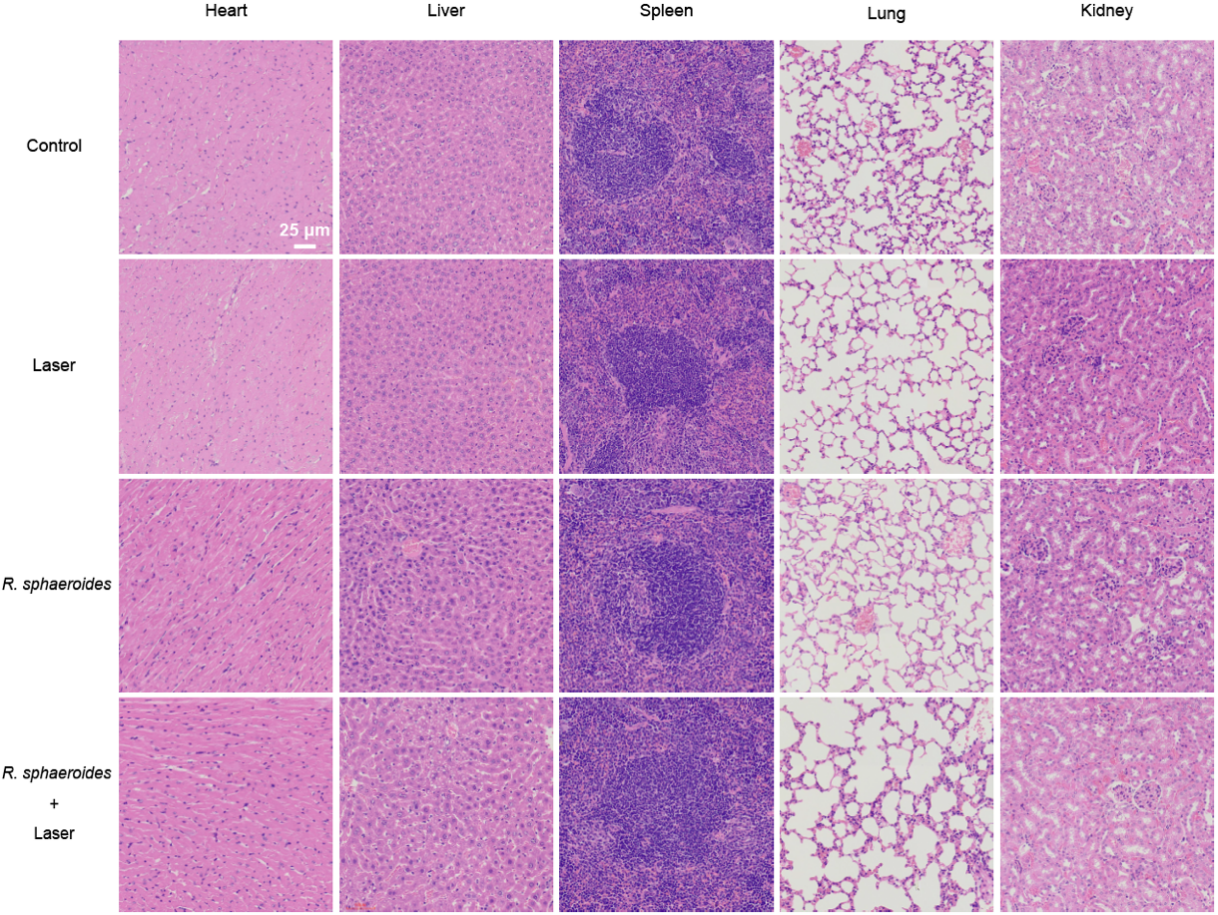
**

**Figure S19.** Histological examination of main organs (heart, liver, spleen, lung and kidney) from mice treated with the PBS group (Control), the only laser control, the only *R. sphaeroides* control, and the *R. sphaeroides* + laser group. Tissues were stained using the Hematoxylin and Eosin (H&E) staining method to assess any histopathological changes. Scale bars, 25 μm.

**Table S1.** The weight changes of BALB/c healthy mice intravenously injected with bacteria at different concentrations and PBS (*n* = 5 biologically independent animals).

| **Group** | **Body weight (g)** | |
| --- | --- | --- |
|  | **Strat (0 d)** | **End (21 d)** |
| 0 CFU/mL | 16.95 ± 1.05 | 19.73 ± 0.66 |
| 1.8×10^9^ CFU/mL | 16.90 ± 1.03 | 19.96 ± 1.69 |
| 3.6×10^9^ CFU/mL | 17.30 ± 1.86 | 20.01 ± 1.31 |
| 7.4×10^9^ CFU/mL | 17.17 ± 0.69 | 19.94 ± 0.51 |
